# A drug repurposing study identifies novel FOXM1 inhibitors with in vitro activity against breast cancer cells

**DOI:** 10.1007/s12032-024-02427-0

**Published:** 2024-06-25

**Authors:** Khaled A. N. Abusharkh, Ferah Comert Onder, Venhar Çınar, Zuhal Hamurcu, Bulent Ozpolat, Mehmet Ay

**Affiliations:** 1 Department of Chemistry, School of Graduate Studies, Çanakkale Onsekiz Mart University, 17020 Çanakkale, Türkiye; 2 Department of Chemistry, Faculty of Science, Natural Products and Drug Research Laboratory, Çanakkale Onsekiz Mart University, 17020 Çanakkale, Türkiye; 3 Department of Chemistry and Chemical Technology, Faculty of Science and Technology, Al-Quds University, Jerusalem, 20002 Palestine; 4 Department of Medical Biology, Faculty of Medicine, Çanakkale Onsekiz Mart University, 17020 Çanakkale, Türkiye; 5 Department of Medical Biology, Faculty of Medicine, Erciyes University, 38039 Kayseri, Türkiye; 6 Department of Nanomedicine, Houston Methodist Research Institute, Houston, TX 77030 USA

**Keywords:** FOXM1, Breast cancer, Triple-negative breast cancer, Drug repurposing, Rabeprazole, Pantoprazole, Molecular docking, Pharmacophore, Targeted therapy, Cell cytotoxicity assay, FOXM1-siRNA transfection, Western blot

## Abstract

FOXM1, a proto-oncogenic transcription factor, plays a critical role in cancer development and treatment resistance in cancers, particularly in breast cancer. Thus, this study aimed to identify potential FOXM1 inhibitors through computational screening of drug databases, followed by in vitro validation of their inhibitory activity against breast cancer cells. In silico studies involved pharmacophore modeling using the FOXM1 inhibitor, FDI-6, followed by virtual screening of DrugBank and Selleckchem databases. The selected drugs were prepared for molecular docking, and the crystal structure of FOXM1 was pre-processed for docking simulations. In vitro studies included MTT assays to assess cytotoxicity, and Western blot analysis to evaluate protein expression levels. Our study identified Pantoprazole and Rabeprazole as potential FOXM1 inhibitors through in silico screening and molecular docking. Molecular dynamics simulations confirmed stable interactions of these drugs with FOXM1. In vitro experiments showed both Pantoprazole and Rabeprazole exhibited strong FOXM1 inhibition at effective concentrations and that showed inhibition of cell proliferation. Rabeprazole showed the inhibitor activity at 10 µM in BT-20 and MCF-7 cell lines. Pantoprazole exhibited FOXM1 inhibition at 30 µM and in BT-20 cells and at 70 µM in MCF-7 cells, respectively. Our current study provides the first evidence that Rabeprazole and Pantoprazole can bind to FOXM1 and inhibit its activity and downstream signaling, including eEF2K and pEF2, in breast cancer cells. These findings indicate that rabeprazole and pantoprazole inhibit FOXM1 and breast cancer cell proliferation, and they can be used for FOXM1-targeted therapy in breast or other cancers driven by FOXM1.

**Graphical Abstract:**

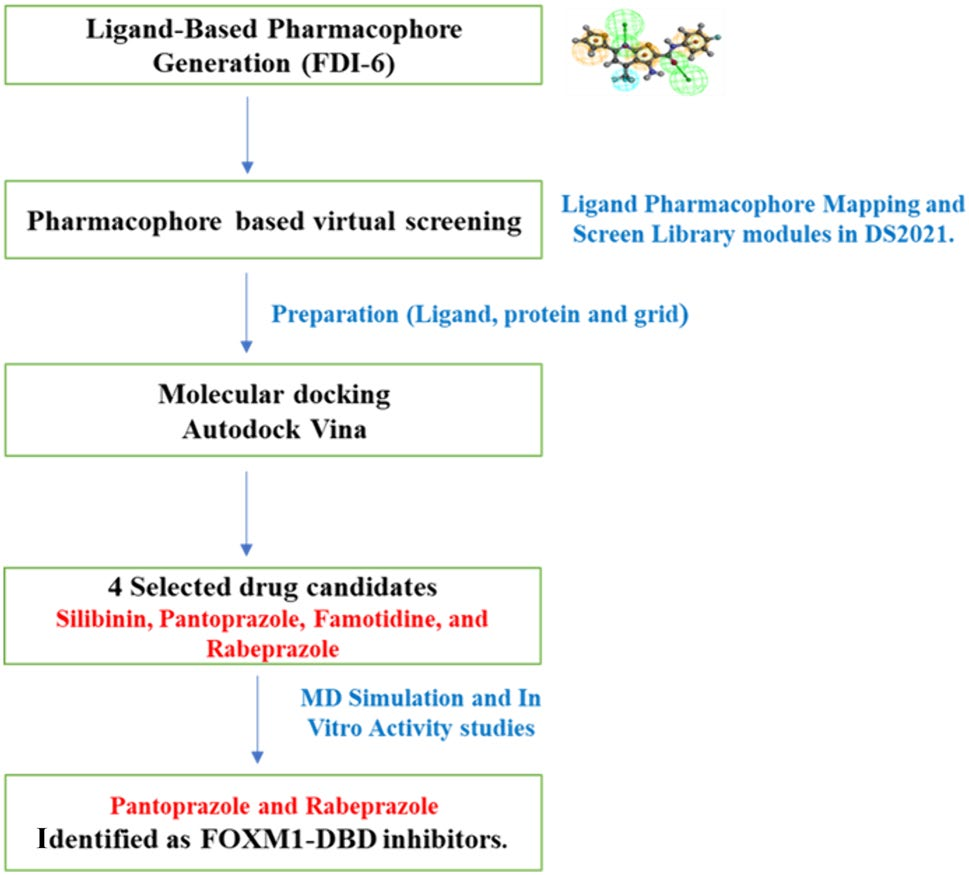

**Supplementary Information:**

The online version contains supplementary material available at 10.1007/s12032-024-02427-0.

## Introduction

Breast cancer is the most commonly diagnosed cancer in women and the second leading cause of cancer-related death [1, 2]. Triple-negative breast cancer (TNBC) accounts for approximately 10–20% of all breast cancers and is characterized by the absence of estrogen receptor (ER), progesterone receptor (PR), and human epidermal growth factor receptor 2 (HER2) expression. This subtype of breast cancer is known for its heterogeneous phenotypic structure, aggressive clinical course, and highly invasive nature, leading to early metastasis and poor prognosis. TNBC patients have shorter survival rates compared to those with other breast cancer subtypes, largely due to its resistance to current treatments and the lack of effective targeted therapies [3–6]. Although a new targeted treatment strategy, Sacituzumab, a Trop-2-receptor-targeted antibody conjugated chemodrug (topoisomerase inhibitor) for metastatic TNBC, was approved by the Food and Drug Administration (FDA) in 2020, and the overall response rate remains low with a median duration of response of only 7.7 months. Immunotherapy has limited effect only a small fraction of patients. Consequently, there is an urgent need to identify new molecular targets and develop effective therapeutic strategies to improve prognosis and poor survival rates in TNBC patients [5, 7].

FOXM1 is a proto-oncogenic transcription factor that belongs to the Forkhead box (FOX) family and plays critical roles in regulating cycle progression, DNA damage response, apoptosis, angiogenesis, and metastasis and tumorigenesis [7, 8]. Dysregulation of FOXM1 has been linked to numerous human diseases, including various cancers, making it an attractive target for therapeutic applications [9, 10]. FOXM1 is overexpression of in TNBC breast cancer and associated with tumor aggressiveness, metastasis, poor patient outcomes, and reduced overall survival rates [4, 8].

The transcriptional activity of FOXM1 is tightly regulated and suppressed by p53 tumor suppressor protein, and p53 mutations detected in about 80% of TNBC patients lead to upregulation of FOXM1 and uncontrolled cell proliferation, migration, invasion, drug resistance, tumor growth, and progression in breast and other cancers [11, 12]. The increased activity of FOXM1 in TNBC contributes to multiple aspects of tumor progression and therapy resistance. Genetic targeting of FOXM1 by RNAi-based strategies have been shown to suppress TNBC tumor growth in mice [13], suggesting it as a promising candidate for targeted therapies in TNBC [13, 14]. Given the multifaceted functions in cancer progression, targeting FOXM1 has emerged as a promising therapeutic approach. In recent years, the development of FOXM1 inhibitors has gained a significant attention as a potential strategy to disrupt the aberrant activity of FOXM1 in breast and other cancers [15, 16].

Identification of pharmacological inhibitors of FOXM1 has been challenging. However, several small-molecule inhibitors have been developed to directly interact with FOXM1-DNA-binding domain (DBD), disrupting its DNA-binding activity, preventing its binding to the promoters of the target genes [17, 18]. Approaches such as inhibition of expression or activity of upstream regulators of FOXM1, such as kinases or coactivators, that indirectly contribute to inhibition of its transcriptional activity has been also used development of potential FOXM1 inhibitors [17, 19, 20]. Furthermore, indirect FOXM1 inhibitors such as natural compounds (i.e., thiostrepton, honokiol, siomycin A) and direct inhibitors (i.e., SR-T100, FDI-6, RCM-1 and DFS lignan, XST-119) have been identified, but none of these inhibitors has advanced to clinical trials [13, 20, 21]. Although these FOXM1 inhibitors are promising candidates often they are not specific and exhibit off-target effects toward other targets. For instance, thiostrepton is a potent FOXM1 inhibitor but it is a highly promiscuous molecule with off-target effects, including inhibition of proteasome and arrest of mitochondrial protein synthesis.

In the last decade, drug repurposing has emerged as a promising strategy and has become an increasingly successful and popular approach for developing new cancer therapies, leveraging existing FDA-approved compounds for novel therapeutic applications [22]. This approach offers several advantages over traditional drug discovery, including reduced time and cost [22]. On the other hand, the conventional drug discovery process is not only time-consuming but also has high failure rates [22, 23]. Therefore, computational approaches such as pharmacophore modeling, molecular docking, and molecular dynamics (MD) studies have significantly gained interest in finding effective candidates for secondary uses of approved drugs for the treatment of related diseases, such as cancer [23].

In the current study, we aimed to identify potential FOXM1 inhibitors through a drug repurposing strategy and employed an integrated approach, including in silico screening of FDA-approved drugs and molecular docking and MD simulation studies. Our studies identified two hit candidates, rabeprazole and pantoprazole, as inhibitors of FOXM1. Rabeprazole and pantoprazole are FDA-approved drugs used to prevent acid secretion from the stomach by acting as proton pump inhibitors primarily used for the treatment of acid-related gastrointestinal disorders such as gastric and duodenal ulcers. Our findings showed that Trp308, Ser290, Arg286, Asn283, His287, and Asn288 amino acid residues on the FOXM1 protein interact with rabeprazole and pantoprazole exhibit strong binding affinity. Both drugs inhibited cell proliferation and FOXM1 protein in breast cancer cell lines in a dose-dependent manner, with rabeprazole being more potent compared to pantoprazole. Our data suggest that rabeprazole and pantoprazole can inhibit FOXM1 and provide a targeted therapeutic option for breast cancer patients and other cancers with aberrant FOXM1 expression, laying the foundation for developing FOXM1-targeted personalized treatment approaches in future.

## Materials and methods

### In silico studies

#### Generation of the ligand-based pharmacophore model

To create a pharmacophore model, we used previously published FOXM1 inhibitor FDI-6 [17, 24]. The features of pharmacophore were determined using the BIOVIA Discovery Studio 2021 (DS 2021) Auto Pharmacophore Generation module [25–27]. This pharmacophore model was used as a three-dimensional (3D) query to search for matching hits from approved drug databases.

#### Pharmacophore-based virtual screening

Pharmacophore-based virtual screening was conducted utilizing the DrugBank (https://go.drugbank.com/) and drug libraries sourced from various categories including anticancer, antidiabetic, anti-infection, and antiparasitic compounds available at (https://www.selleckchem.com). The pharmacophore model derived from FDI-6 was employed to screen these databases using the Ligand Pharmacophore Mapping and Screen Library modules within DS [27, 28].

#### Ligand preparation

Among the mapped and best screened results following pharmacophore-based screening study, the FDA-approved drugs were downloaded as.sdf format to prepare for molecular docking studies. The downloaded compounds in.sdf format were converted to.pdb files using Open Babel software (version 2.3.2) [29]. The ligand structures were then prepared in AutoDock Tools (ADT) from MGL software packages (version 1.5.6), where the Gustier partial charge was assigned and were saved in rigid format [30]. Subsequently, all structures were transformed into the.pdbqt format, enabling their utilization in subsequent investigations.

#### Protein and grid preparation

The crystal structure of FOXM1 protein was downloaded from the protein data bank (PDB) (3G73) [28, 31]. The crystal structures of the protein were subjected to pre-processing steps to optimize the docking experiment. First, all hetero atoms, water molecules, and chain A, C, and D were eliminated from the crystal structures. Subsequently, only polar hydrogen atoms were added to the remaining protein structures [31, 32]. The resulting protein structures were then saved in the.pdbqt format, ensuring compatibility for subsequent docking simulations.

#### Active site prediction

Grid preparation was performed using AutoDock Tools version 1.5.7 protocols [31, 33–35]. The literature related to FOXM1-DNA-binding domain (DBD) was thoroughly examined to identify the binding site residues. As a result, the specific residues crucial for FOXM1–DNA interaction, namely Asn283, Arg286, and His287, were determined. These residues were found to play essential roles in facilitating the interaction between FOXM1 and DNA [28, 31]. The grid box for the binding site of the FOXM1-DBD amino acids, where the ligand molecule binds, was determined.

#### Molecular docking studies

Molecular docking was carried out using AutoDock Vina [31, 32, 35–37] with the selected approved drugs. Subsequently, the top hit drugs were determined based on their docking scores and then, the two-dimensional (2D) ligand interactions were analyzed using the DS.

#### Molecular dynamics (MD) simulations

The dynamic behavior of the protein–ligand complex was investigated through MD simulations using the Desmond of Schrodinger [31]. The Protein Preparation Wizard was used to prepare the FOXM1 protein. To mimic physiological conditions, the complex was immersed in a solvated environment consisting of an orthorhombic periodic box filled with simple point charge (SPC) water. Neutralization was achieved by adding 150mM NaCl solution. The solvated structure was then subjected to energy minimization and restrained to its initial location using the OPLS2005 force field [26, 27, 38, 39]. Subsequently, 100ns MD simulations were carried out under NPT ensemble at a temperature of 300 K and a pressure of 1 atm. The observed protein–ligand interactions were obtained during simulations. These analyses provided insights into the behavior and dynamics of the protein–ligand complex in the simulated environment.

### In vitro activity studies

#### Cell lines and culture conditions

Human TNBC cells (BT-20) and estrogen receptor-positive (ER +) breast cancer cells (MCF-7) were purchased from the American Type Culture Collection (Manassas, VA, USA). All breast cancer cell lines were cultured in Dulbecco’s Modified Eagle’s Medium (DMEM)/F12 supplemented with 10% fetal bovine serum (Sigma-Aldrich, St. Louis, MO). These cells were cultured under standard conditions at 37 °C and 5% CO_2_ in DMEM-F12 (1:1) supplemented with 10% fetal bovine serum (FBS) and 1% penicillin/streptomycin (PS) [40, 41].

#### Cell cytotoxicity assay (MTT assay)

To determine the cell cytotoxicity, the drugs were dissolved in DMSO and then were applied to the cells in the ranging concentrations from 5 µM to 100 µM for 96 h. Untreated cells and DMSO-treated cells were used as a control. After the indicated time periods, MTT solution (3-(4,5-dimethylthiazol-2-yl)-2,5-diphenyltetrazolium bromide) was added to each well and the cells were incubated for 3 h at 37 °C in a CO_2_ incubator [41]. The plates were measured at 570 nm using a Thermo Spectrophotometer. The data are represented using GraphPad Prism 7 program. The percentage of cell viability was determined [39].

#### Transfection with siRNA

Two different small interfering RNAs (siRNAs) targeting FOXM1 gene (FOXM1#1 (SASI_Hs01_00052108), FOXM1#2 (SASI_Hs01_00243977), and non-silencing control siRNAs (WD00909801)) were purchased from Sigma-Aldrich. Exponentially growing breast cancer cells were plated and 24 h later transfected with FOXM1 siRNAs or control siRNA at a final concentration of 50 nM for 72 h using HiPerFect Transfection Reagent (Qiagen) according to the manufacturer’s protocol. The concentrations of siRNAs were chosen based on previous studies [6, 8, 40, 42, 43]. Non-silencing control siRNA-transfected cells were used as controls. After treatment, the cells were harvested and processed for western analysis.

#### Western blotting

Western blot analysis was carried out according to our previously reported studies [40–42]. TNBC (BT-20) and ER + (MCF-7) cell lines were treated with the indicated concentrations of Rabeprazole and Pantoprazole. Additionally, both MCF-7 and BT-20 cells were transfected with siRNAs (50 nM) for 72 h. Following siRNA treatment, cells were harvested, washed with PBS, and lysed using lysis buffer containing phosphatase, protease, and 1% NaCl. Protein concentrations were determined using a BCA assay kit. The proteins were separated by SDS-PAGE gel electrophoresis, transferred to membranes, and probed with primary antibodies against FOXM1, eEF2, p-EF2 (Thr56) (Cell Signaling Technologies), eEF2K (Proteintech), and β-actin (Proteintech). After washing with TBS-T, the membranes were incubated with anti-rabbit and anti-mouse secondary antibodies. Finally, protein bands were visualized using an Alpha Imager [41].

## Results and discussion

### In silico studies

#### Virtual screening and hit drug identification

**Fig. 1:**
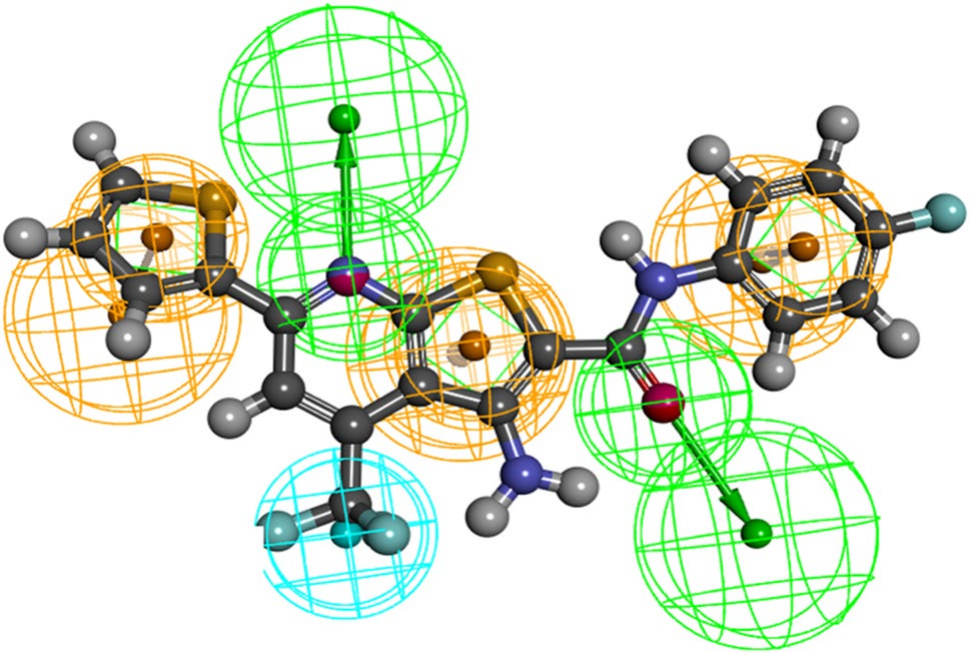
Pharmacophore features of FDI-6 generated by DS. The hydrogen bond acceptors, aromatic features, and hydrophobic are displayed in mesh spheres of green, orange, and blue, respectively. The arrows indicate the constraint direction (Color figure online)

To identify the hits that bind to DBD of FOXM1, we used the pharmacophore features of FDI-6 as a positive control, as depicted in (Fig. 1). The pharmacophore model consists of six distinct pharmacophore features as illustrated in (Fig. 1). Among these features, two hydrogen bond acceptor features are associated with the nitrogen atoms of the thieno[2,3-b]pyridine ring, while another hydrogen bond acceptor feature corresponds to the carbonyl group. Additionally, three aromatic ring features represent the phenyl ring attached to the NH group, the thieno[2,3-b]pyridine ring, and the thiophene ring. Finally, a hydrophobic group is centered around the CF_3_ group. This comprehensive description of the pharmacophore model provides valuable insights into the structural requirements necessary for ligand binding and interaction within the FOXM1-DBD. We employed a 3D query for virtual screening of the DrugBank (approximately 10,000 compounds) and Selleckchem databases (10,439 compounds). Virtual screening was conducted using Pharmacophore Mapping and Screen Library protocols within the DS software. The screen library protocol was configured to retrieve the top 20 hits, resulting in the identification of 391 compounds (185 from DrugBank and 206 from Selleckchem). Subsequently, 11 compounds were chosen as hits, primarily based on their FDA approval status, along with considerations of fit value, drug’s chemical properties, and structural similarity to FDI-6.

Based on previously published studies, it is well established that three conserved amino acid residues, including Asn283, Arg286, and His287, play crucial roles in the FOXM1 protein-DNA interaction [28, 31, 36, 44]. His287 establishes direct and indirect hydrogen bonds with the DNA base, mediated by a water molecule [28, 44]. Perturbation of this hydrogen bonding network, possibly due to a His287 mutation, reduces the binding affinity of FOXM1 to DNA. Furthermore, Asn283 exhibits two conformations: vertical, engaging in two hydrogen bonds with DNA, and horizontal, forms minimal DNA interactions but engages in two hydrogen bonds with Arg286. Mutations in Asn283 decrease DNA-binding affinity of FOXM1, while mutations in Arg286 abolish its DNA hydrogen binding, potentially favoring the vertical conformation of Asn283 and enhancing its DNA interactions [28, 31, 36, 44]. Understanding the structural and functional significance of these amino acid residues sheds light on the mechanism of FOXM1-DNA binding and its implications in cellular processes governed by FOXM1.

#### Molecular docking studies

Based on an assessment of FDA approval status, fit values, drug chemical properties, and structural similarity to FDI-6, we identified eleven potential drugs (hits) among the FDA-approved drugs. Subsequently, molecular docking was performed using AutoDock Vina, followed by molecular docking analysis in the DS, to evaluate the binding affinity of the selected compounds. Each of the eleven potential candidates underwent docking and their respective docking scores and fit values are documented in (Table 1.) These hits exhibited molecular interactions with critical amino acids (His287, Arg286, and Asn283) that play a role in inhibiting FOXM1-DBD. These findings underscore the potential of these selected compounds as promising candidates for further evaluation in the context of binding to and inhibiting FOXM1 function.

**Table 1:** Docking scores and fit values of the selected 11 drugs targeting the FOXM1-DBD

No	Drugs (ID)	Database	Fit value	Method (discovery studio)	Docking score (kcal/mol)
1	Lifitegrast (S3714)	Selleckchem	1.44037	Ligand pharmacophore mapping	− 6.3
2	**Silibinin (*S2357*)**	**Selleckchem**	**3.39885**	**Ligand pharmacophore mapping**	**− 6.1**
3	**Pantoprazole (S2105)**	**Selleckchem**	**3.87575**	**Screen library/best results**	**− 5.9**
4	Sulfasalazine (S1576)	Selleckchem	3.57656	Screen library/best results	− 5.9
5	Ilaprazole (S3666)	Selleckchem	3.67673	Screen library/best results	− 5.6
6	**Rabeprazole (S4845)**	**Selleckchem**	**3.75232**	**Screen library/best results**	**− 5.5**
7	Cardarine-GW501516 (DB05416)	DrugBank	3.31021	Screen library/best results	− 5.5
8	GW0742 (S8020)	Selleckchem	3.58484	Ligand Pharmacophore mapping	− 5.5
9	Phthalylsulfathiazole (S5700)	Selleckchem	3.78803	Screen library/best results	− 5.3
10	Sulfadimethoxine (DB06150)	DrugBank	3.88599	Screen library/best results	− 5.0
11	**Famotidine (DB00927)**	**DrugBank**	**3.37979**	**Screen library/best results**	**− 4.5**
12	**FDI-6 (reference inhibitor)**	**–**	**–**	**–**	**− 5.6**

The selected drugs for in vitro studies were highlighted in bold

**Fig. 2:**
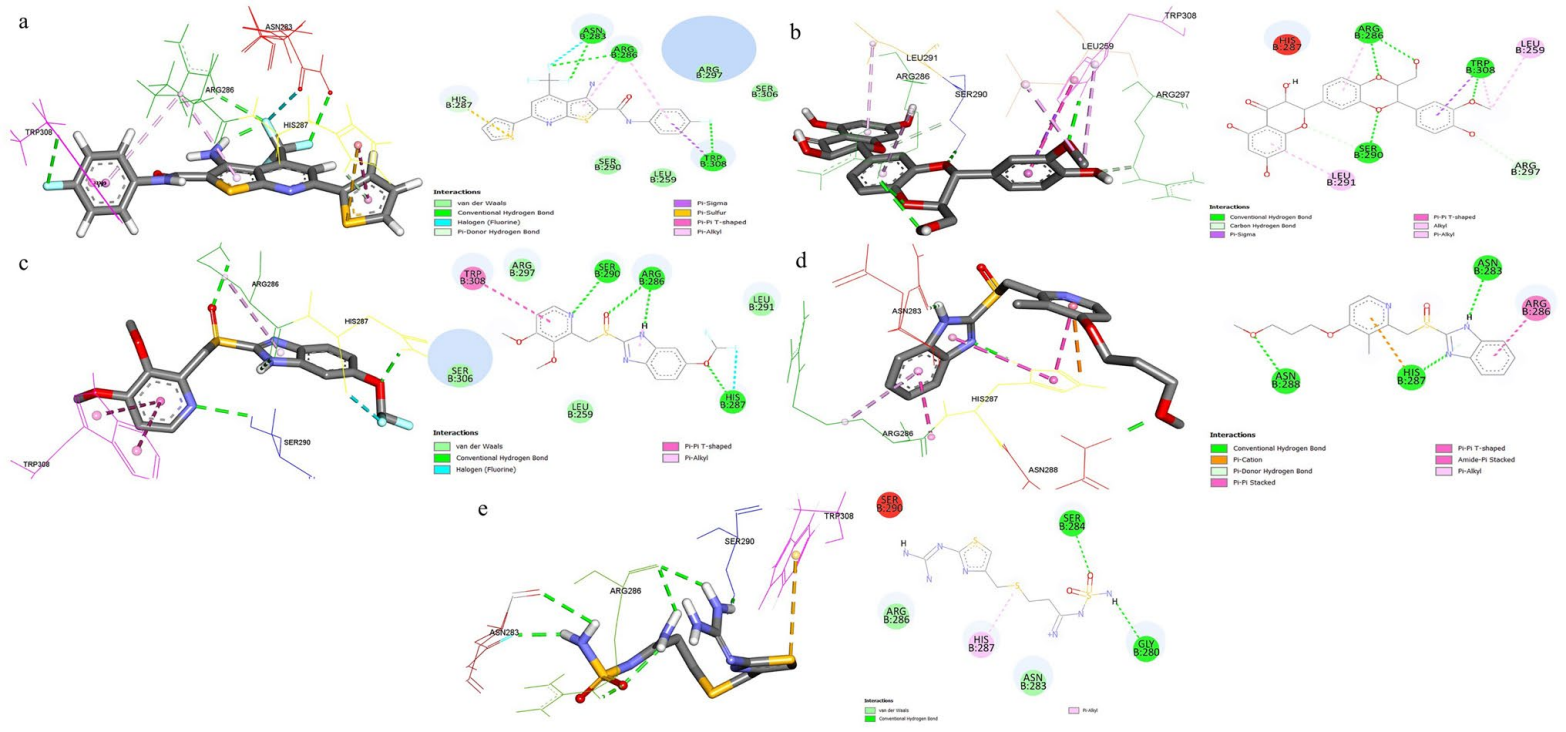
The key interactions of the drugs with FOXM1-DBD; the 2D structural representation of FOXM1-DBD residues and 3D representation showing the docked hits interacting with the binding site residues of FOXM1-DBD. **a** FDI-6, **b** Silibinin, **c** Pantoprazole, **d** Rabeprazole, and **e** Famotidine

**Fig. 3:**
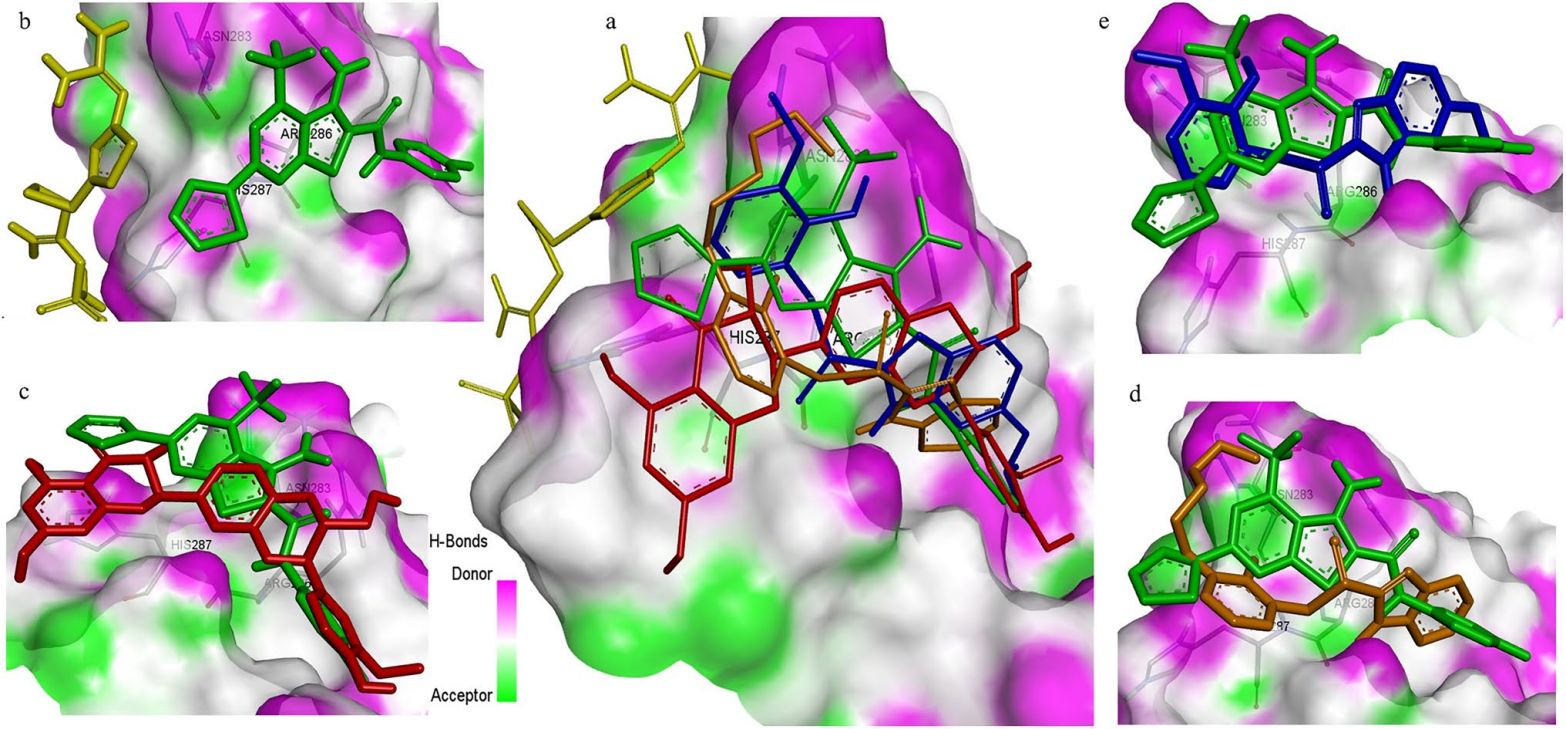
The comparative docking poses of Pantoprazole (blue), Silibinin (red), Famotidine (yellow), and Rabeprazole (orange) with FDI-6 (green) within the active site of FOXM1-DBD. **a** The docking poses of all selected drugs alongside FDI-6 in FOXM1-DBD and individual comparisons, depicting the docking poses of Famotidine (**b**), Silibinin (**c**), Rabeprazole (**d**), and Pantoprazole (**e**), with FDI-6 in the active site of FOXM1-DBD (Color figure online)

Based on the molecular docking scores and ligand/drug interaction diagrams (Fig. 2, 3), four potential drugs were identified, including Silibinin, Famotidine, Pantoprazole, and Rabeprazole. Notably, Silibinin and Pantoprazole exhibited higher binding energies of − 6.1 kcal/mol and − 5.9 kcal/mol, respectively, compared to the positive control and reference compound FDI-6 (− 5.6 kcal/mol). On the other hand, Rabeprazole and Famotidine displayed lower binding energies compared to the positive control (− 5.5 kcal/mol, − 4.5 kcal/mol, respectively). The docking results for the selected drugs and standard FDI-6 are summarized in (Table 1).

#### Interaction of selected compounds with active site residues on FOXM1

The interactions of selected candidates including Pantoprazole, Rabeprazole, Famotidine, and natural compound Silibinin with the active site residues of FOXM1-DBD were analyzed and compared with the positive control FDI-6. FDI-6 interacted with His287, Asn283, Arg286, and Trp308 through hydrophobic interactions and conventional hydrogen bonds, as shown in (Fig. 2a). Pantoprazole interacted with Trp308, Ser290, Arg286, and His287, while Rabeprazole interacted with Asn288, His287, Asn283, and Arg286. Pantoprazole formed four hydrogen bonds, as shown in (Fig. 2c), and Rabeprazole exhibited various interactions as depicted in (Fig. 2d). Famotidine interacted with Ser284, Giy280, and His287, forming two conventional hydrogen bonds and a pi-sulfur bond, as shown in (Fig. 2e). Silibinin showed the interactions with Arg286, Ser290, Trp308, Leu291, Arg297, and Leu259 forming four conventional hydrogen bonds (Fig. 2b)*.* These findings highlight the specific residues involved in the binding interactions of the inhibitors with FOXM1-DBD providing insights into the molecular interactions and potential mechanisms of inhibition.

Analysis of the interactions between the selected inhibitors and the active site residues of FOXM1-DBD revealed that the standard inhibitor FDI-6 interacts with the critical amino acids His287, Asn283, and Arg286 through two conventional hydrogen bonds. Rabeprazole also displayed the same interaction pattern, forming three conventional hydrogen bonds with these important amino acids. This indicates that Rabeprazole exhibits a stronger interaction with the target compared to the positive control FDI-6. These findings from our in silico studies suggest that Rabeprazole is a more potent inhibitor of FOXM1-DBD than FDI-6.

Pantoprazole exhibited notable interactions with two critical amino acids His287 and Arg286 forming three conventional hydrogen bonds. Notably, it showed superior binding compared to FDI-6 and like rabeprazole, it did not interact with Asn283. This finding highlights the FOXM1-DBD inhibitory potential of Pantoprazole. On the other hand, Silibinin formed two conventional hydrogen bonds both with Arg286, while Famotidine displayed weaker binding by engaging in only one hydrophobic interaction with the amino acid His287. The analysis of the interactions between the selected drugs and FDI-6 showed that Pantoprazole and Rabeprazole interact more strongly with critical amino acids in FOXM1 compared to FDI-6. These findings provide valuable insights into the structural basis of inhibitor–FOXM1–-DBD interactions and suggest Rabeprazole and Pantoprazole as promising candidates for testing in in vitro studies for the inhibition of FOXM1-DBD. Next, we aimed to elucidate the potential binding modes of the selected hits with FOXM1-DBD and to compare their binding modes with that of the standard inhibitor FDI-6 (Fig. 3a).

Silibinin which exhibited interactions with a single critical amino acid for target inhibition demonstrated a binding mode relatively similar to that of FDI-6 (Fig. 3c). Rabeprazole, interacting with all essential amino acids for target inhibition, demonstrated coordination modes comparable to those observed with FDI-6 (Fig. 3d). However, Famotidine displayed a significantly different binding mode compared to FDI-6, suggesting a completely different binding mode (Fig. 3b). Pantoprazole, interacting with two critical amino acids, shared coordination modes like those observed in FDI-6 (Fig. 3e).

The common coordination modes between Rabeprazole and Pantoprazole with regard to FDI-6 suggest their potential as novel FOXM1 inhibitors. Moreover, a relatively close association binding mode exhibited by Silibinin in comparison with FDI-6, underscores its distinct binding characteristics. In contrast, the unique binding model displayed by Famotidine, differing from that of FDI-6, emphasizes its divergent binding properties. These findings elucidate the binding dynamics between the selected drugs and FOXM1, offering valuable insights into the development of novel inhibitors targeting this critical protein.

Based on the studies in the literature, FDI-6 is a potent inhibitor of FOXM1 and acts by disrupting the interaction of FOXM1 with target gene promoters, leading to decreased transcriptional activity [31] By binding to the DNA-binding domain DBD in which it targets critical amino acids such as, His287, Asn283, and Arg286, has been shown be effective in preclinical studies [17, 24, 31]. In vitro preclinical TNBC models, FDI-6 has demonstrated significant antitumor effects, including inhibition of cell proliferation, induction of apoptosis, and suppression of migration and invasion [17, 31]. FDI-6 also sensitizes TNBC cells to chemotherapy, potentially overcoming treatment resistance [17, 31]. However, the safety profile of FDI-6 has not been documented.

In a recent study conducted in 2022 by Zhang et al. [28], the inhibitory potential of XST-20 on the FOXM1-DBD was demonstrated in ovarian cancer cells. Notably, XST-20 demonstrated promising binding interactions with key amino acids, such as His287, Asn283, and Arg286 within the FOXM1-DBD, indicating that this compound also holds significant importance as a FOXM1 inhibitor. However, preclinical in vivo studies demonstrating its in efficacy in tumor models in mice are needed to pave the way for potential clinical applications targeting FOXM1 in various cancers.

#### MD simulation studies

**Fig. 4:**
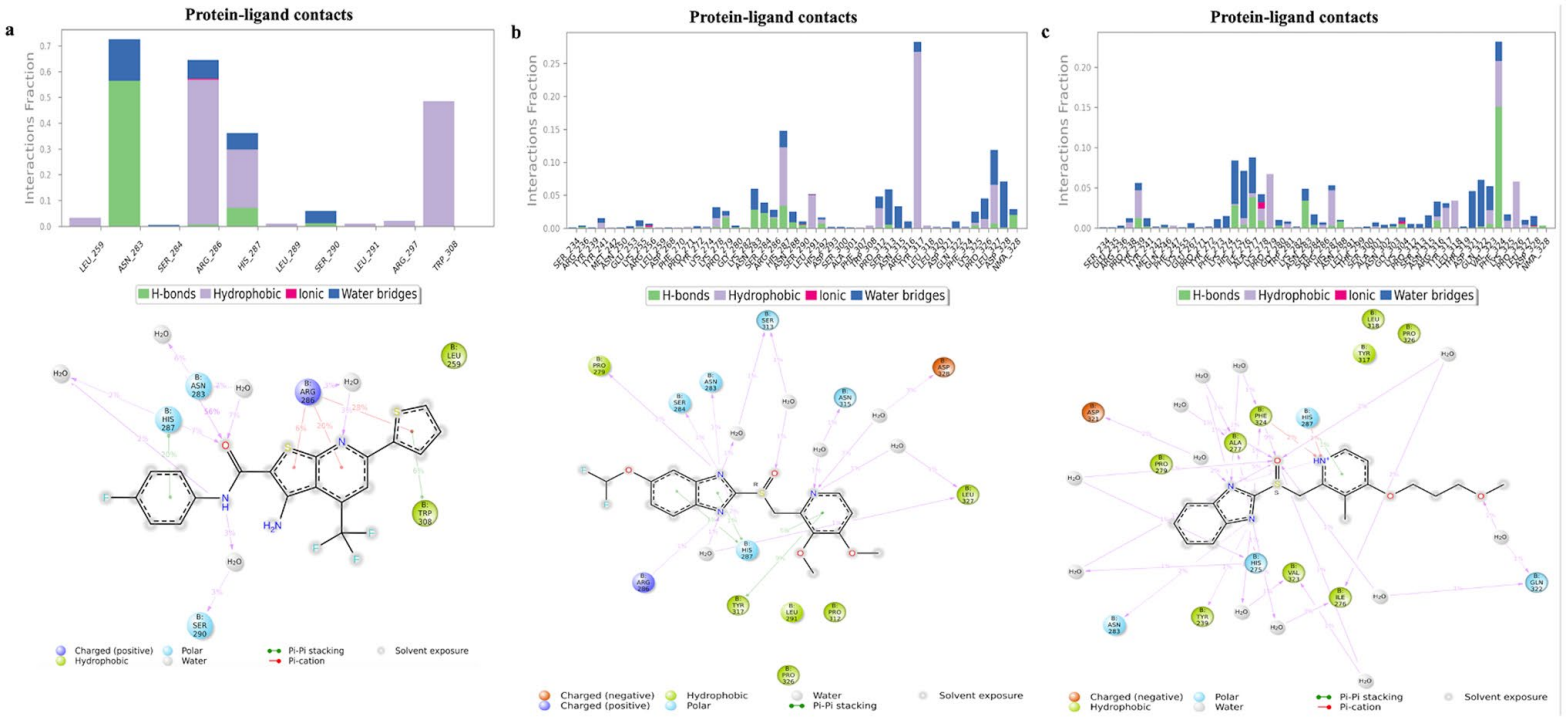
MD Simulation analysis of protein–ligand (drug) interactions and contact plot with residues on FOXM1 protein for **a** FDI-6, **b** Pantoprazole, and **c** Rabeprazole

Throughout the MD simulations of the protein-hit complexes, we conducted a comprehensive analysis of the possible interactions of Rabeprazole and Pantoprazole. The top docking pose and the best conformation interactions with FOXM1 were utilized. The average root-mean-square deviation (RMSD) value was approximately 3.70 Å for the FOXM1-DBD-FDI-6 complex (Fig. S1a) Supplementary Information), suggesting a moderate degree of structural deviation from the initial conformation during the MD simulation. This indicates that while the complex undergoes some structural fluctuations, it maintains a relatively stable overall conformation throughout the simulation. The RMSF plot provides additional insight into the dynamic behavior of individual amino acid residues within the complex, illustrating their varying degrees of mobility over the course of the simulation. Analyzing the RMSF (Fig. S1b) plot enables a detailed examination of regions within the protein that experience significant structural flexibility or rigidity, which can further advance our understanding of the functional dynamics of the FOXM1-DBD-ligand (i.e., FDI-6) complex. In the FOXM1/FDI-6 complex (Fig. 4a), significant hydrogen bond interactions were observed between the ligand and the amino acid residues of Asn283 and His287, as well as Ser290 and Arg286, indicating that these hydrogen bonds contribute to the stability and binding affinity of the complex. Furthermore, hydrophobic interactions involving Trp308 and positively charged atom interactions mediated by the backbone interactions of Arg286. These hydrophobic and electrostatic interactions play a crucial role in the overall structure and function of the complex.

In the complex involving Pantoprazole (Fig. 4b), a network of hydrogen bond interactions was identified among the ligand and Asn283, Ser284, Arg286, and His287, which play critical roles in establishing specific contacts and fostering the stability of the complex. Additionally, side chain interactions involving Ser313, Asn315, Asp328, and Leu327 were observed, contributing to the overall binding affinity of the complex. Furthermore, the hydrophobic interactions with Pro279, Leu327, and Tyr317 were noted, thereby augmenting the stability and interactions within the complex. Polar molecular interactions involving His287, Asn283, Ser284, Asn315, and Ser313 were found to bolster the overall structural integrity and specificity of the complex. The average root-mean-square deviation (RMSD) value of approximately 3.30 Å for the FOXM1-DBD-Pantoprazole complex (Fig. S1c) indicates a moderate degree of structural deviation from the initial conformation during the molecular dynamics’ simulation, suggesting a relatively stable overall conformation. The root-mean-square fluctuation (RMSF) (Fig. S1d) plot provided additional insights into the dynamic behavior of individual amino acid residues within the complex, elucidating their varying degrees of mobility throughout the simulation. Analyzing the RMSF plot facilitates a comprehensive examination of regions within the protein experiencing notable structural flexibility or rigidity, thereby enhancing our understanding of the functional dynamics of the FOXM1-DBD-Pantoprazole complex.

In the complex with Rabeprazole and FOXM1 (Fig. 4c), we identified hydrogen bond interactions between Ala277, Phe324, Tyr239, and Asn283. These hydrogen bonds play a significant role in the formation and stabilization of the complex. Moreover, side chain interactions involving Asp321, Gln322, Ile276, Val323, and His275 were observed, contributing to the overall binding affinity and specificity of the complex. The hydrophobic interactions with Ala277, Phe324, Tyr239, Val323, and Ile276 were also detected, further strengthening the stability and interactions within the complex. We also identified negatively charged atom interactions, mediated by the backbone interactions to Asp321 be involved in the complex formation. Polar molecular interactions involving His287, Asn283, Gln322, and His275 were identified, providing additional stability and specificity to the complex. The average root-mean-square deviation (RMSD) value was detected as approximately 2.30 Å for the FOXM1-DBD-Rabeprazole complex (Fig. S1e) indicates a moderate degree of structural deviation from the initial conformation during the MD simulation, suggesting a relatively stable overall conformation. The root-mean-square fluctuation (RMSF) (Fig. S1f) plot provided additional insights into the dynamic behavior of individual amino acid residues within the complex, elucidating their varying degrees of mobility throughout the simulation. Analyzing the RMSF plot facilitates a comprehensive examination of regions within the protein experiencing notable structural flexibility or rigidity, thereby enhancing our understanding of the functional dynamics of the FOXM1-DBD-Rabeprazole complex.

These detailed findings shed light on the molecular interactions within the protein–ligand complexes, uncovering key features that contribute to their potential as inhibitors of FOXM1-DBD. Understanding these interactions is crucial for rational drug design and optimization. The information obtained from the MD simulations can guide future studies aiming to develop novel and potent inhibitors targeting FOXM1-DBD for cancer treatment. Further investigations, including experimental validation and structure–activity relationship studies, are warranted to fully explore the therapeutic potential of these compounds.

### In vitro activity

#### Selected drugs reduced cell proliferation and viability of breast cancer cells

To evaluate the effects of selected drugs on breast cancer cell line proliferation and viability, we investigated their impact on (TNBC) BT-20, and estrogen receptor-positive (ER +) MCF-7 cell lines using the MTT cell viability assay. Cells were exposed to varying concentrations of Pantoprazole, Famotidine, Rabeprazole, and Silibinin for 96 h and their viability was evaluated through MTT assays.

**Fig. 5:**
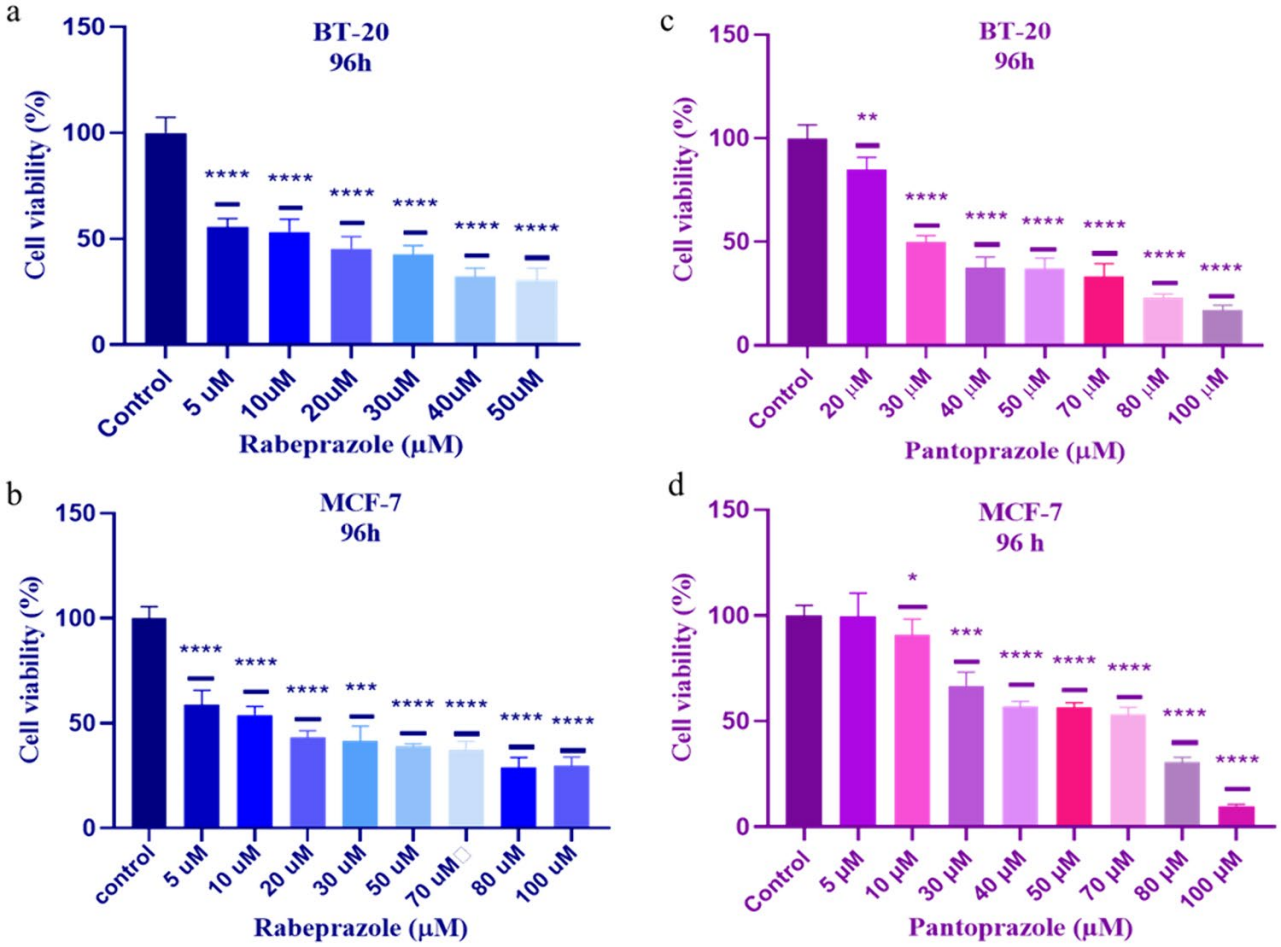
Rabeprazole and pantoprazole inhibit breast cancer cell proliferation. The effects of Rabeprazole and Pantoprazole were evaluated by MTT cell viability assay after 96 h of treatment of BT-20 and MCF-7 breast cancer cells. Rabeprazole and Pantoprazole reduced the cell viability on BT-20 (**a**, **c**) and MCF-7 (**b**, **d**) cells. Data were presented as means ± S.D. *p < 0.05, **p < 0.01, ***p < 0.001, and ****p < 0.0001

Our findings demonstrated that Rabeprazole significantly affected the viability of BT-20 and MCF-7 cells at concentrations ranging between 5 µM and 20 µM (Fig. 5a, b). Pantoprazole exhibited significantly affected cell proliferation and viability at 30 µM and 70 µM in BT-20 and MCF-7 cell lines, respectively (Fig. 5c, d). However, Famotidine and Silibinin did not induce cytotoxic effects within the tested concentration range (5–100 µM). Consequently, Silibinin and Famotidine were excluded from western blot analysis due to their limited effects on cancer cells at high concentrations compared to Rabeprazole and Pantoprazole.

#### Rabeprazole and pantoprazole inhibit FOXM1 in breast cancer cells

**Fig. 6:**
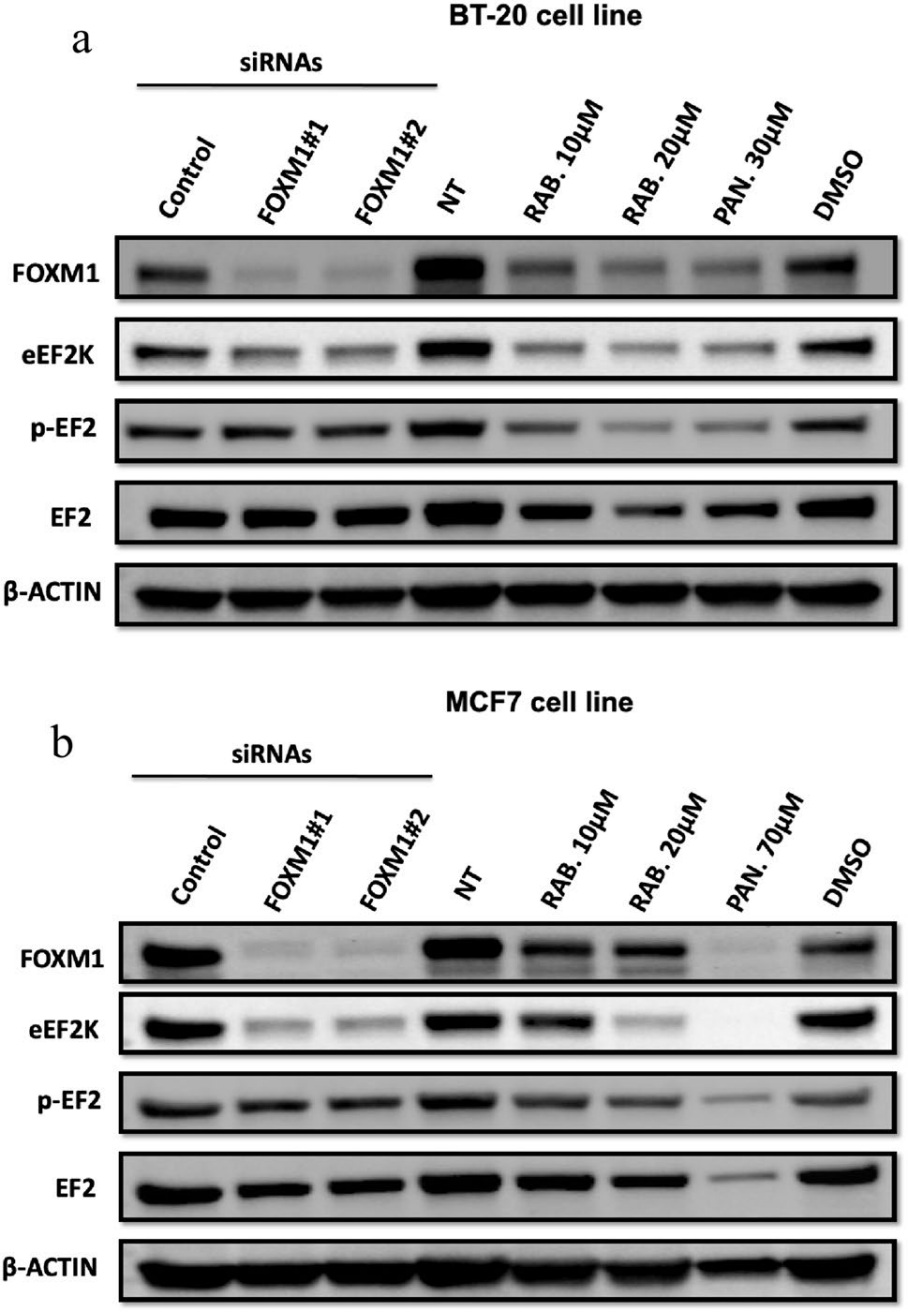
Rabeprazole and pantoprazole inhibit FOXM1 in breast cancer cells. The cells were treated with siRNA FOXM1#1 or FOXM1#2 or control siRNAs (50 nM, 72 h); the cells were treated with Rabeprazole and Pantoprazole or DMSO control vehicle. Rabeprazole and Pantoprazole inhibit FOXM1 expression in TNBC BT-20 (**a**) and MCF-7 (**b**) cells. Protein extracts were isolated 72 h after treatments and β-Actin was used as a loading control. *RAB* rabeprazole, *PAN* pantoprazole

The inhibitor activity of the two drugs against FOXM1 was determined in BT-20 and MCF-7 cell lines. For this purpose, we evaluated FOXM1 levels after treatments with Rabeprazole and Pantoprazole in the cell lines at various concentrations. Both drugs exhibited strong FOXM1 inhibition at effective concentrations that showed inhibition of cell proliferation. Rabeprazole showed the inhibitor activity at 10 µM in BT-20 and MCF-7 cell lines (Fig. 6a, b). Pantoprazole exhibited FOXM1 inhibition at 30 µM and 70 µM in BT-20 and MCF-7 cells, respectively (Fig. 6a, b). These results indicate that Rabeprazole is the most potent inhibitor against FOXM1 in both cancer cell lines. Previously, we have shown that TNBC cells (MDA-MB-231, BT-20) have higher FOXM1 expression than other breast cancer cell lines, including the MCF-7 cells [43]. Therefore, TNBC cells, BT-20 are expected to be more responsive to Pantoprazole compared to MCF-7 cells response to Pantoprazole. We have previously reported that FOXM1 transcriptionally drives expression of elongation factor 2 kinase (eEF2K), which promotes cell proliferation and growth TNBC tumor xenografts in mice [42]. FOXM1 inhibition leads to downregulation of eEF2K and suppresses cell proliferation and tumor growth in TNBC models [42, 45]. Therefore, we investigated the inhibitor effect of two drugs on eEF2K expression that is the downstream mediator of FOXM1 [43]. The results showed that the treatment of cells with both drugs reduced levels of eEF2K and its direct downstream target p-EF2 (Thr56) in (TNBC) BT-20 and (ER +) MCF-7 cell lines.

To confirm whether Rabeprazole and Pantoprazole suppress FOXM1 and its downstream target eEF2K expressions, we also genetically silenced FOXM1 gene using a specific siRNA targeting FOXM1 mRNA in two different breast cancer cells and evaluated expressions of FOXM1 and eEF2K by Western blot analysis. As expected, knockdown of FOXM1 by siRNA decreased the FOXM1 and its downstream targets eEF2K expression as well as phosphorylation of EF2 (p-EF2) at Thr56 in MCF-7 and BT-20 cells compared to control treatments. These studies suggested that genetic and pharmacological inhibition of FOXM1 by siRNA, Rabeprazole, and Pantoprazole, respectively, suppress expression of FOXM1 in both BT-20 and MCF-7 cells (Fig. 6a, b). In this analysis, β-actin was used as a loading control. Overall, these results indicate that the Rabeprazole and Pantoprazole treatments inhibit FOXM1 in breast cancer cells and these drugs may be used as promising FOXM1 anticancer agents for targeting FOXM1 in solid tumors.

Pantoprazole and Rabeprazole are the FDA-approved anti-acid proton pump inhibitors (PPIs) primarily used for the treatment of acid-related gastrointestinal disorders and ulcers to reduce acid secretion from the stomach. While their main indication is not cancer treatment, there is increasing interest in exploring their potential roles in cancer management [46–48].

Pantoprazole has been shown to exert antiproliferative effects in gastric cancer, leukemic cancer, colorectal cancer, and pancreatic cancer [46–48], inhibit tumor growth, induce apoptosis, and enhance the effectiveness of chemotherapy drugs [46]. Pantoprazole has been shown to inhibit proteasomal degradation [49]. Similarly, Rabeprazole has been shown to demonstrate anticancer properties in gastric cancer, colorectal cancer, and esophageal cancer [50]. Rabeprazole inhibits tumor growth and the expression of cancer-promoting genes and sensitize cancer cells to chemotherapy [50]. However, the mechanism of action of the antiproliferative effects of these drugs was not fully understood.

We have previously demonstrated that FOXM1 is overexpressed in TNBC cells and higher expression of FOXM1 is associated with shorter survival and worse prognosis of patients [6, 8, 42, 43]. Furthermore, we showed for the first time that FOXM1 gene using siRNA suppressed TNBC tumor growth in mice and validated it as a molecular target for TNBC [6, 8, 42, 43]. Our current study provides the first evidence that Pantoprazole and Rabeprazole can bind to FOXM1 and inhibit its activity and downstream signaling including eEF2K and pEF2 in breast cancer cells. As expected, inhibition of FOXM1 with Pantoprazole and Rabeprazole leads to significant reduced of cell proliferation and survival in two different breast cancer cells.

Overall, our studies also shed light on the anticancer effects of two anti-acid drugs Pantoprazole and Rabeprazole. While further research is needed to fully elucidate the potential of Pantoprazole and Rabeprazole in cancer treatment, these findings highlight the potential for repurposing these commonly used PPIs for oncological purposes [46, 49].

#### Structure–activity relationship (SAR)

**Fig. 7:**
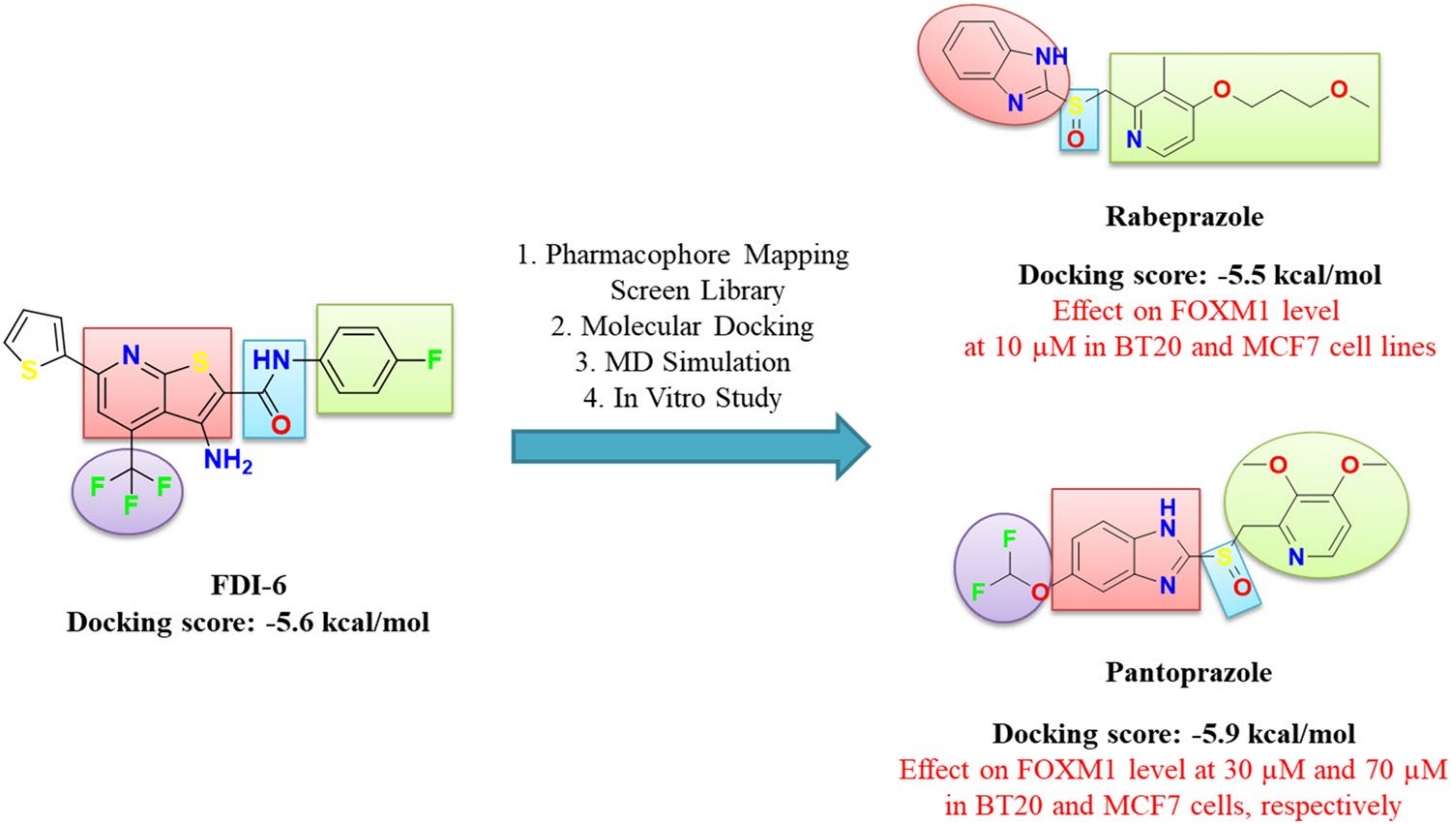
An overview for future perspectives

According to our combined in silico and in vitro results, we analyzed the chemical structures of reference molecule FDI-6 and approved drugs. A schematic overview of this study is shown in (Fig. 7). Although reference molecule has thieno[2,3-b] pyridine scaffold, the studied drugs contribute to FOXM1 activity with a benzimidazole ring. Furthermore, the sulfinyl group of the approved drugs could take the place of amide substitution in FDI-6. Rabeprazole decreased the FOXM1 activity levels in Western blot analysis due to the pyridine ring bearing long ethylene groups (-CH_2_-). A difluoromethoxy group at 5-position on benzimidazole scaffold of Pantoprazole may contribute to the activity more than a trifluoromethane substituent at 4-position on FDI-6 reference molecule. In addition, pyridine ring including various substituents can be used in the further design of new analogues of FDI-6 in place of substituted phenyl ring bearing an electron-withdrawing fluorine atom. As a result, we demonstrate that the benzimidazole scaffold and *O*-substituted pyridine ring can be used in place of the thieno[2,3-b] pyridine scaffold of FDI-6.

## Conclusion

Our study utilizing pharmacophore-based virtual screening and molecular docking analysis identified for the first time that two FDA-approved anti-acid drugs, Pantoprazole and Rabeprazole, as potential FOXM1 inhibitors and that these drugs may be used in cancer therapy for targeting FOXM1-driven cancers, including breast cancer. These compounds demonstrated interaction with key amino acids involved in FOXM1-DBD inhibition, highlighting their mechanism of action as inhibitors. Further analysis demonstrated that Pantoprazole and Rabeprazole interact with active site residues of FOXM1-DBD with distinct binding patterns.

Consequently, Rabeprazole and Pantoprazole are potential novel inhibitors of FOXM1 and may be used as FOXM1-targeted cancer therapy in treatment of breast cancer and other FOXM1-driven cancers. These results contribute to the understanding of FOXM1-mediated cellular processes and may aid in the development of novel treatments for breast cancer and other diseases involving dysregulated FOXM1 activity.

Furthermore, further in vitro and in vivo analyses are needed before these drugs can be used in clinical translation. These scaffolds may contribute to design of new and effective molecules for future perspectives.

### Supplementary Information

Below is the link to the electronic supplementary material.

Supplementary file1 (TIF 5660 KB)

Supplementary file2 (DOCX 4705 KB)

## Data Availability

The datasets generated and analyzed during the current study are included in the manuscript. Additional data are available from the corresponding authors upon reasonable request.

## Abbreviations

2D: Two-dimensional
3D: Three-dimensional
ADME: Absorption, distribution, metabolism, and excretion
DBD: DNA-binding domain
ER: Estrogen receptor
eEF2K: Eukaryotic elongation factor 2 kinase
FDA: Food and drug administration
FDI-6: Fork domain inhibitor-6
FOXM1: Forkhead box protein M1
FOX: Forkhead box
HER2: Human epidermal growth factor receptor 2
MD: Molecular dynamics
PDB: Protein data bank
pEF2: Phospho-eukaryotic elongation factor 2
RMSF: Root-mean-square fluctuation
RMSD: Root-mean-square deviation
SPC: Simple point charge
TNBC: Triple-negative breast cancer

## References

[1] Bray F, Laversanne M, Sung H, Ferlay J, Siegel RL, Soerjomataram I, Jemal A. Global cancer statistics 2022: GLOBOCAN estimates of incidence and mortality worldwide for 36 cancers in 185 countries. CA Cancer J Clin. 2024;74(3):229–63. 10.3322/caac.21834. 38572751 10.3322/caac.21834

[2] Lyu L, Zhang S, Deng Y, Wang M, Deng X, Yang S, et al. Regulatory mechanisms, functions, and clinical significance of CircRNAs in triple-negative breast cancer. J Hematol Oncol. 2021;14(1):41. 10.1186/s13045-021-01052-y. 33676555 10.1186/s13045-021-01052-y PMC7937293

[3] Narrandes S, Huang S, Murphy L, Xu W. The exploration of contrasting pathways in triple negative breast cancer (TNBC). BMC Cancer. 2018;18(1):22. 10.1186/s12885-017-3939-4. 29301506 10.1186/s12885-017-3939-4 PMC5753474

[4] Guo W, Lin L, He X, He F, Wang C, Chen N, et al. Biomarkers of DNA repair and related pathways: significance of treatment in triple-negative breast cancer. Crit Rev Oncog. 2017;22(5–6):427–37. 10.1615/CritRevOncog.2017020575. 29604922 10.1615/CritRevOncog.2017020575

[5] Abba MC, Lacunza E, Butti M, Aldaz CM. Breast cancer biomarker discovery in the functional genomic age: a systematic review of 42 gene expression signatures. Biomark Insights. 2010;5:103–18. 10.4137/BMI.S5740. 21082037 10.4137/BMI.S5740 PMC2978930

[6] Bayraktar R, Ivan C, Bayraktar E, Kanlikilicer P, Kabil NN, Kahraman N, et al. Dual suppressive effect of miR-34a on the FOXM1/eEF2-kinase axis regulates triple-negative breast cancer growth and invasion. Clin Cancer Res. 2018;24(17):4225–41. 10.1158/1078-0432.CCR-17-1959. 29748184 10.1158/1078-0432.CCR-17-1959

[7] Wang X, Kiyokawa H, Dennewitz MB, Costa RH. The forkhead Box m1b transcription factor is essential for hepatocyte DNA replication and mitosis during mouse liver regeneration. Proc Natl Acad Sci. 2002;99(26):16881–6. 10.1073/pnas.252570299. 12482952 10.1073/pnas.252570299 PMC139238

[8] Hamurcu Z, Delibaşı N, Nalbantoglu U, Sener EF, Nurdinov N, Tascı B, et al. FOXM1 plays a role in autophagy by transcriptionally regulating beclin-1 and LC3 genes in human triple-negative breast cancer cells. J Mol Med. 2019;97(4):491–508. 10.1007/s00109-019-01750-8. 30729279 10.1007/s00109-019-01750-8

[9] Wang Y, Zhou X, Xu M, Weng W, Zhang Q, Yang Y, Wei P, Du X. OTUB1-catalyzed deubiquitination of FOXM1 facilitates tumor progression and predicts a poor prognosis in ovarian cancer. Oncotarget. 2016;7(24):36681–97. 10.18632/oncotarget.9160. 27167337 10.18632/oncotarget.9160 PMC5095031

[10] Xu N, Jia D, Chen W, Wang H, Liu F, Ge H, et al. FoxM1 is associated with poor prognosis of non-small cell lung cancer patients through promoting tumor metastasis. PLoS ONE. 2013;8(3):e59412. 10.1371/journal.pone.0059412. 23536876 10.1371/journal.pone.0059412 PMC3607616

[11] Madureira PA, Varshochi R, Constantinidou D, Francis RE, Coombes RC, Yao K-M, et al. The forkhead box M1 protein regulates the transcription of the estrogen receptor α in breast cancer cells. J Biol Chem. 2006;281(35):25167–76. 10.1074/jbc.M603906200. 16809346 10.1074/jbc.M603906200

[12] Tan Y, Raychaudhuri P, Costa RH. Chk2 mediates stabilization of the FoxM1 transcription factor to stimulate expression of DNA repair genes. Mol Cell Biol. 2007;27(3):1007–16. 10.1128/MCB.01068-06. 17101782 10.1128/MCB.01068-06 PMC1800696

[13] Song X, Fiati Kenston SS, Zhao J, Yang D, Gu Y. Roles of FoxM1 in cell regulation and breast cancer targeting therapy. Med Oncol. 2017;34(3):41. 10.1007/s12032-017-0888-3. 28176242 10.1007/s12032-017-0888-3

[14] Chen T, Xiong J, Yang C, Shan L, Tan G, Yu L, et al. Silencing of FOXM1 transcription factor expression by adenovirus-mediated RNA interference inhibits human hepatocellular carcinoma growth. Cancer Gene Ther. 2014;21(3):133–8. 10.1038/cgt.2014.8. 24577129 10.1038/cgt.2014.8

[15] Yan C, Higgins PJ. Drugging the undruggable: transcription therapy for cancer. Biochimica et Biophysica Acta (BBA)—Rev Cancer. 2013;1835(1):76–85. 10.1016/j.bbcan.2012.11.002. 10.1016/j.bbcan.2012.11.002 PMC3529832 23147197

[16] Uddin S, Ahmed M, Hussain A, Abubaker J, Al-Sanea N, AbdulJabbar A, et al. Genome-wide expression analysis of middle eastern colorectal cancer reveals FOXM1 as a novel target for cancer therapy. Am J Pathol. 2011;178(2):537–47. 10.1016/j.ajpath.2010.10.020. 21281787 10.1016/j.ajpath.2010.10.020 PMC3070566

[17] Gormally MV, Dexheimer TS, Marsico G, Sanders DA, Lowe C, Matak-Vinković D, et al. Suppression of the FOXM1 transcriptional programme via novel small molecule inhibition. Nat Commun. 2014;5:5165. 10.1038/ncomms6165. 25387393 10.1038/ncomms6165 PMC4258842

[18] Kalin TV, Wang I-C, Ackerson TJ, Major ML, Detrisac CJ, Kalinichenko VV, et al. Increased levels of the FoxM1 transcription factor accelerate development and progression of prostate carcinomas in both TRAMP and LADY transgenic mice. Cancer Res. 2006;66(3):1712–20. 10.1158/0008-5472.CAN-05-3138. 16452231 10.1158/0008-5472.CAN-05-3138 PMC1363687

[19] Gartel AL. A new target for proteasome inhibitors: FoxM1. Expert Opin Investig Drugs. 2010;19(2):235–42. 10.1517/13543780903563364. 20074015 10.1517/13543780903563364 PMC3532816

[20] Hegde NS, Sanders DA, Rodriguez R, Balasubramanian S. The transcription factor FOXM1 is a cellular target of the natural product thiostrepton. Nat Chem. 2011;3(9):725–31. 10.1038/nchem.1114. 21860463 10.1038/nchem.1114

[21] Raychaudhuri P, Park HJ. FoxM1: a master regulator of tumor metastasis. Cancer Res. 2011;71(13):4329–33. 10.1158/0008-5472.CAN-11-0640. 21712406 10.1158/0008-5472.CAN-11-0640 PMC3129416

[22] Sleire L, Førde HE, Netland IA, Leiss L, Skeie BS, Enger PØ. Drug repurposing in cancer. Pharmacol Res. 2017;124:74–91. 10.1016/j.phrs.2017.07.013. 28712971 10.1016/j.phrs.2017.07.013

[23] Lu S-H, Wu JW, Liu H-L, Zhao J-H, Liu K-T, Chuang C-K, et al. The discovery of potential acetylcholinesterase inhibitors: a combination of pharmacophore modeling, virtual screening, and molecular docking studies. J Biomed Sci. 2011;18(1):8. 10.1186/1423-0127-18-8. 21251245 10.1186/1423-0127-18-8 PMC3036604

[24] Ulhaka K, Kanokwiroon K, Khongkow M, Bissanum R, Khunpitak T, Khongkow P. The anticancer effects of FDI-6, a FOXM1 inhibitor, on triple negative breast cancer. Int J Mol Sci. 2021;22(13):6685. 10.3390/ijms22136685. 34206484 10.3390/ijms22136685 PMC8269391

[25] Matada GS, Dhiwar PS, Abbas N, Singh E, Ghara A, Patil R, Raghavendra NM. Pharmacophore modeling, virtual screening, molecular docking and dynamics studies for the discovery of HER2-tyrosine kinase inhibitors: an in-silico approach. J Mol Struct. 2022;5(1257):132531. 10.1016/j.molstruc.2022.132531

[26] Kalin S, Comert OF. Discovery of potential RSK1 inhibitors for cancer therapy using virtual screening, molecular docking, molecular dynamics simulation, and MM/GBSA calculations. J Biomol Struct Dyn. 2023. 10.1080/07391102.2023.2291830. 38084766 10.1080/07391102.2023.2291830

[27] Erdoğan M, Comert OF. Synthesis, anticancer activity and molecular modeling study of novel substituted triazole linked tetrafluoronaphthalene hybrid derivatives. J Biomol Struct Dyn. 2023. 10.1080/07391102.2023.2252914. 37676264 10.1080/07391102.2023.2252914

[28] Zhang Z, Xue S, Gao Y, Li Y, Zhou Z, Wang J, et al. Small molecule targeting FOXM1 DNA binding domain exhibits anti-tumor activity in ovarian cancer. Cell Death Discov. 2022;8(1):280. 10.1038/s41420-022-01070-w. 35680842 10.1038/s41420-022-01070-w PMC9184618

[29] O’Boyle NM, Banck M, James CA, Morley C, Vandermeersch T, Hutchison GR. Open Babel: an open chemical toolbox. J Cheminform. 2011;3:33. 10.1186/1758-2946-3-33. 21982300 10.1186/1758-2946-3-33 PMC3198950

[30] Morris GM, Huey R, Lindstrom W, Sanner MF, Belew RK, Goodsell DS, et al. AutoDock4 and AutoDockTools4: automated docking with selective receptor flexibility. J Comput Chem. 2009;30(16):2785–91. 10.1002/jcc.21256. 19399780 10.1002/jcc.21256 PMC2760638

[31] Luo G, Lin X, Vega-Medina A, Xiao M, Li G, Wei H, et al. Targeting of the FOXM1 Oncoprotein by E3 ligase-assisted degradation. J Med Chem. 2021;64(23):17098–114. 10.1021/acs.jmedchem.1c01069. 34812040 10.1021/acs.jmedchem.1c01069

[32] Tabatabaei Dakhili SA, Pérez DJ, Gopal K, Tabatabaei Dakhili SY, Ussher JR, Velázquez-Martínez CA. A structure-activity relationship study of forkhead domain inhibitors (FDI): the importance of halogen binding interactions. Bioorg Chem. 2019;93:103269. 10.1016/j.bioorg.2019.103269. 31654840 10.1016/j.bioorg.2019.103269

[33] Rasul HO, Aziz BK, Ghafour DD, Kivrak A. In silico molecular docking and dynamic simulation of eugenol compounds against breast cancer. J Mol Model. 2022;28(1):17. 10.1007/s00894-021-05010-w. 10.1007/s00894-021-05010-w 34962586

[34] Yasmin T, Ali MT, Haque S, Hossain M. Interaction of quercetin of onion with axon guidance protein receptor, NRP-1 plays important role in cancer treatment: an in silico approach. Interdiscip Sci. 2017;9(2):184–91. 10.1007/s12539-015-0137-4. 26706905 10.1007/s12539-015-0137-4

[35] Chen Y, Ruben EA, Rajadas J, Teng NNH. In silico investigation of FOXM1 binding and novel inhibitors in epithelial ovarian cancer. Bioorg Med Chem. 2015;23(15):4576–82. 10.1016/j.bmc.2015.06.002. 26164623 10.1016/j.bmc.2015.06.002

[36] Tabatabaei-Dakhili SA, Aguayo-Ortiz R, Domínguez L, Velázquez-Martínez CA. Untying the knot of transcription factor druggability: molecular modeling study of FOXM1 inhibitors. J Mol Graph Model. 2018;80:197–210. 10.1016/j.jmgm.2018.01.009. 29414039 10.1016/j.jmgm.2018.01.009

[37] Trott O, Olson AJ. AutoDock Vina: improving the speed and accuracy of docking with a new scoring function, efficient optimization, and multithreading. J Comput Chem. 2010;31(2):455–61. 10.1002/jcc.21334. 19499576 10.1002/jcc.21334 PMC3041641

[38] Jorgensen WL, Tirado-Rives J. The OPLS [optimized potentials for liquid simulations] potential functions for proteins, energy minimizations for crystals of cyclic peptides and crambin. J Am Chem Soc. 1988;110(6):1657–66. 10.1021/ja00214a001. 27557051 10.1021/ja00214a001

[39] Comert Onder F, Sahin N, Davutlar G, Onder A, Ay M. Nitro compounds inhibit breast cancer cell proliferation, migration, and colony formation: molecular docking, molecular dynamics simulations and pharmacological properties. ChemistrySelect. 2023;8:e202304614. 10.1002/slct.202304614. 10.1002/slct.202304614

[40] Cınar V, Hamurcu Z, Guler A, Nurdinov N, Ozpolat B. Serotonin 5-HT7 receptor is a biomarker poor prognostic factor and induces proliferation of triple-negative breast cancer cells through FOXM1. Breast Cancer. 2022;29(6):1106–20. 10.1007/s12282-022-01391-9. 36006564 10.1007/s12282-022-01391-9

[41] Comert Onder F, Kahraman N, Bellur Atici E, Cagir A, Kandemir H, Tatar G, et al. Target-driven design of a coumarinyl chalcone scaffold based novel EF2 kinase inhibitor suppresses breast cancer growth *in vivo*. ACS Pharmacol Transl Sci. 2021;4(2):926–40. 10.1021/acsptsci. 33860211 10.1021/acsptsci PMC8033782

[42] Hamurcu Z, Kahraman N, Ashour A, Ozpolat B. FOXM1 transcriptionally regulates expression of integrin β1 in triple-negative breast cancer. Breast Cancer Res Treat. 2017;163(3):485–93. 10.1007/s10549-017-4207-7. 28361350 10.1007/s10549-017-4207-7

[43] Hamurcu Z, Ashour A, Kahraman N, Ozpolat B. FOXM1 regulates expression of eukaryotic elongation factor 2 kinase and promotes proliferation, invasion and tumorgenesis of human triple negative breast cancer cells. Oncotarget. 2016;7(13):16619–35. 10.18632/oncotarget.7672. 26918606 10.18632/oncotarget.7672 PMC4941339

[44] Littler DR, Alvarez-Fernandez M, Stein A, Hibbert RG, Heidebrecht T, Aloy P, et al. Structure of the FoxM1 DNA-recognition domain bound to a promoter sequence. Nucleic Acids Res. 2010;38(13):4527–38. 10.1093/nar/gkq194. 20360045 10.1093/nar/gkq194 PMC2910063

[45] El-Helby AGA, Sakr H, Eissa IH, Al-Karmalawy AA, El-Adl K. Benzoxazole/benzothiazole-derived VEGFR-2 inhibitors: design, synthesis, molecular docking, and anticancer evaluations. Arch Pharm (Weinheim). 2019;352(12):e1900178. 10.1002/ardp.201900178. 31596514 10.1002/ardp.201900178

[46] Patel A, Spychalski P, Antoszewska M, Regula J, Kobiela J. Proton pump inhibitors and colorectal cancer: a systematic review. World J Gastroenterol. 2021;27(44):7716–33. 10.3748/wjg.v27.i44.7716. 34908809 10.3748/wjg.v27.i44.7716 PMC8641055

[47] Zhang B, Yang Y, Shi X, Liao W, Chen M, Cheng AS-L, et al. Proton pump inhibitor pantoprazole abrogates adriamycin-resistant gastric cancer cell invasiveness via suppression of Akt/GSK-β/β-catenin signaling and epithelial–mesenchymal transition. Cancer Lett. 2015;356:704–12. 10.1016/j.canlet.2014.10.016. 25449432 10.1016/j.canlet.2014.10.016

[48] Feng S, Zheng Z, Feng L, Yang L, Chen Z, Lin Y, et al. Proton pump inhibitor pantoprazole inhibits the proliferation, self-renewal and chemoresistance of gastric cancer stem cells via the EMT/β-catenin pathways. Oncol Rep. 2016;36(6):3207–14. 10.3892/or.2016.5154. 27748935 10.3892/or.2016.5154

[49] Cao Y, Chen M, Tang D, Yan H, Ding X, Zhou F, et al. The proton pump inhibitor pantoprazole disrupts protein degradation systems and sensitizes cancer cells to death under various stresses. Cell Death Dis. 2018;9(6):604. 10.1038/s41419-018-0642-6. 29789637 10.1038/s41419-018-0642-6 PMC5964200

[50] Babu D, Mudiraj A, Yadav N, Y.B.V.K. C, Panigrahi M, Prakash Babu P. Rabeprazole has efficacy per se and reduces resistance to temozolomide in glioma via EMT inhibition. Cell Oncol. 2021;44(4):889–905. 10.1007/s13402-021-00609-w. 10.1007/s13402-021-00609-w PMC12980772 33948872

