# Supplementary figures and images for: A drug repurposing study identifies novel FOXM1 inhibitors with in vitro activity against breast cancer cells
Source: Med Oncol. 2024 Jun 25;41(8):188. doi: 10.1007/s12032-024-02427-0 (PMC11199234; doi:10.1007/s12032-024-02427-0)

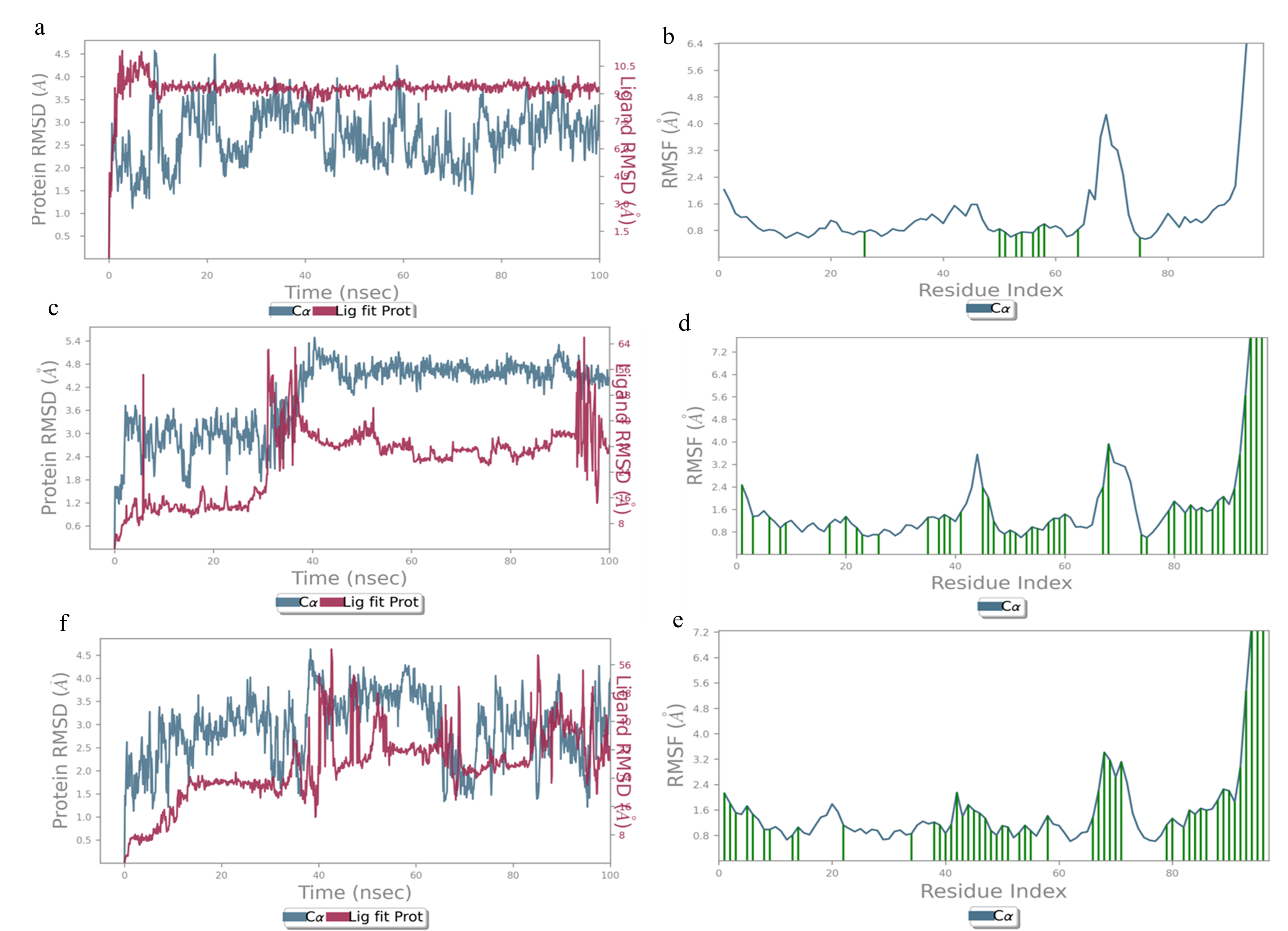

Supplement: Supplementary file 1 — Supplementary file1 (TIF 5660 KB) [file 12032_2024_2427_MOESM1_ESM.tif]
