# Supplementary material for: A drug repurposing study identifies novel FOXM1 inhibitors with in vitro activity against breast cancer cells
Source: Med Oncol. 2024 Jun 25;41(8):188. doi: 10.1007/s12032-024-02427-0 (PMC11199234; doi:10.1007/s12032-024-02427-0)
Supplement: Supplementary file 2 — Supplementary file2 (DOCX 4705 KB) [file 12032_2024_2427_MOESM2_ESM.docx]

**Supplementary Information**

**A drug repurposing study identifies novel FOXM1 inhibitors with *in vitro* activity against breast cancer cells**

Khaled A.N. Abusharkh^1-3^, Ferah Comert Onder*^,4^, Venhar Çınar^5^, Zuhal Hamurcu^5^, Bulent Ozpolat*^,6^, Mehmet Ay*^,2^

^1^Department of Chemistry, School of Graduate Studies, Çanakkale Onsekiz Mart University, Çanakkale 17020, TURKIYE.

^2^Department of Chemistry, Faculty of Science, Natural Products and Drug Research Laboratory, Çanakkale Onsekiz Mart University, 17020, Çanakkale, TURKIYE.

^3^Department of Chemistry and Chemical Technology, Faculty of Science and Technology, Al-Quds University, Jerusalem 20002, PALESTINE.

^4^Department of Medical Biology, Faculty of Medicine, Çanakkale Onsekiz Mart University, 17020, Çanakkale, TURKIYE.

^5^Department of Medical Biology, Faculty of Medicine, Erciyes University, Kayseri 38039, TURKIYE.

^6^Department of Nanomedicine and Methodist Neil Cancer Center-Houston, Houston Methodist Research Institute, Houston, TX 77030, USA.

*Corresponding authors.

E-mail addresses [ferahcomertonder@comu.edu.tr](mailto:ferahcomertonder@comu.edu.tr) (FCO), [bozpolat@houstonmethodist.org](mailto:bozpolat@houstonmethodist.org) (BO), [mehmetay06@comu.edu.tr](mailto:mehmetay06@comu.edu.tr) (MA)


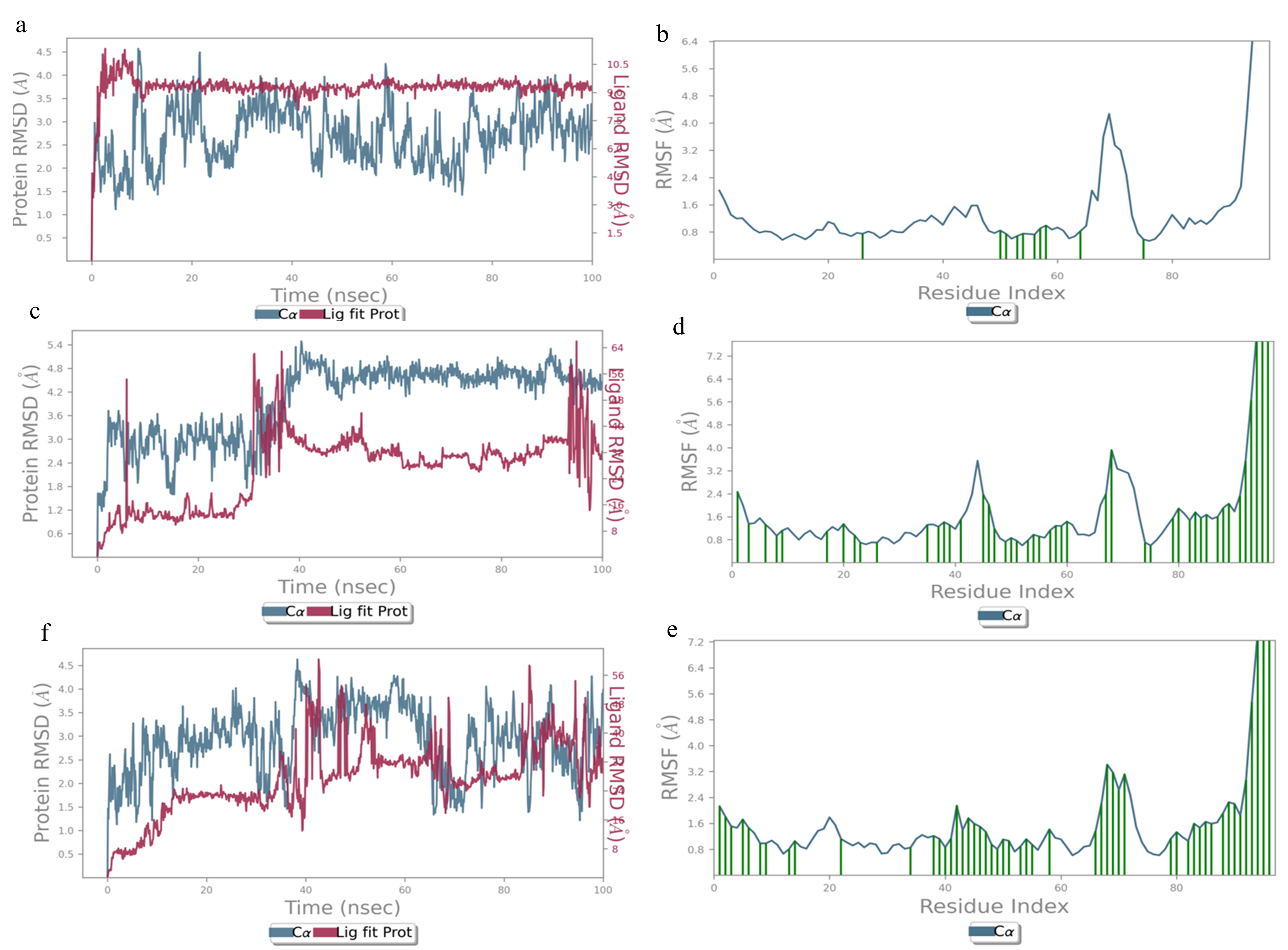


**Fig. S1.** Root Mean Square Deviation (RMSD) and Root Mean Square Fluctuation (RMSF) Analysis of the Known Inhibitor (FDI-6) and Repurposed Drugs: (a, b) FDI-6, (c, d) Rabeprazole, and (e, f) Pantoprazole.

**Original western blot bands**


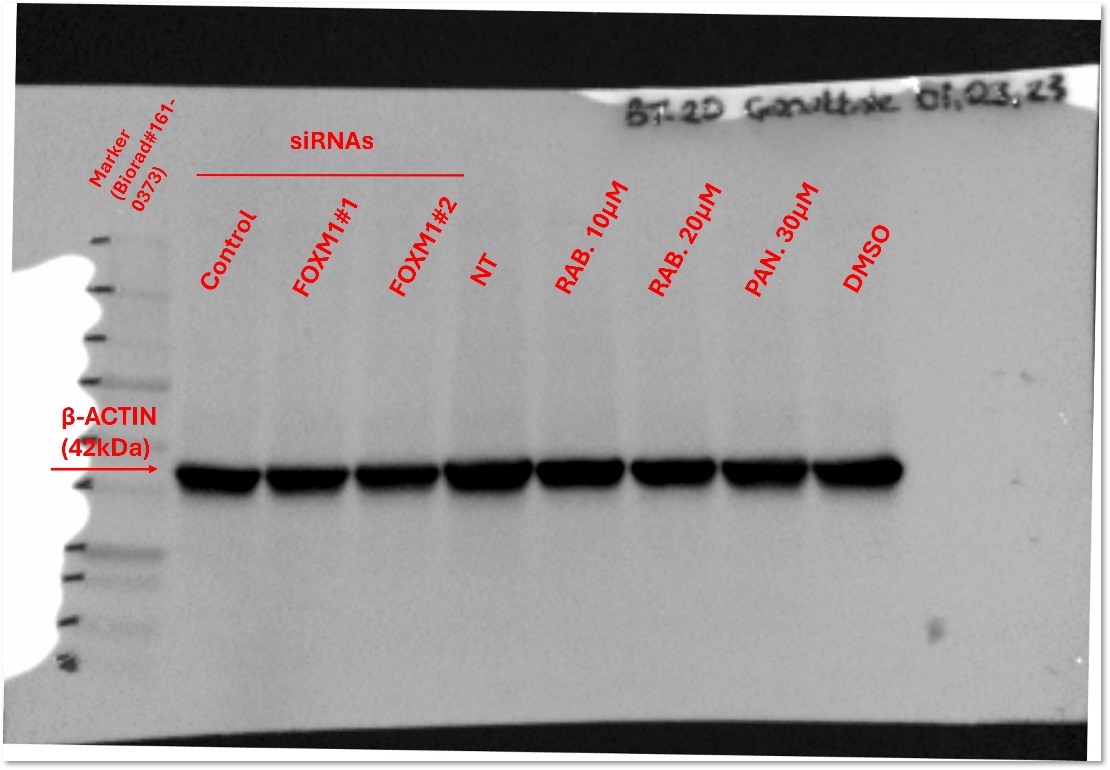


BT-20 β-actin


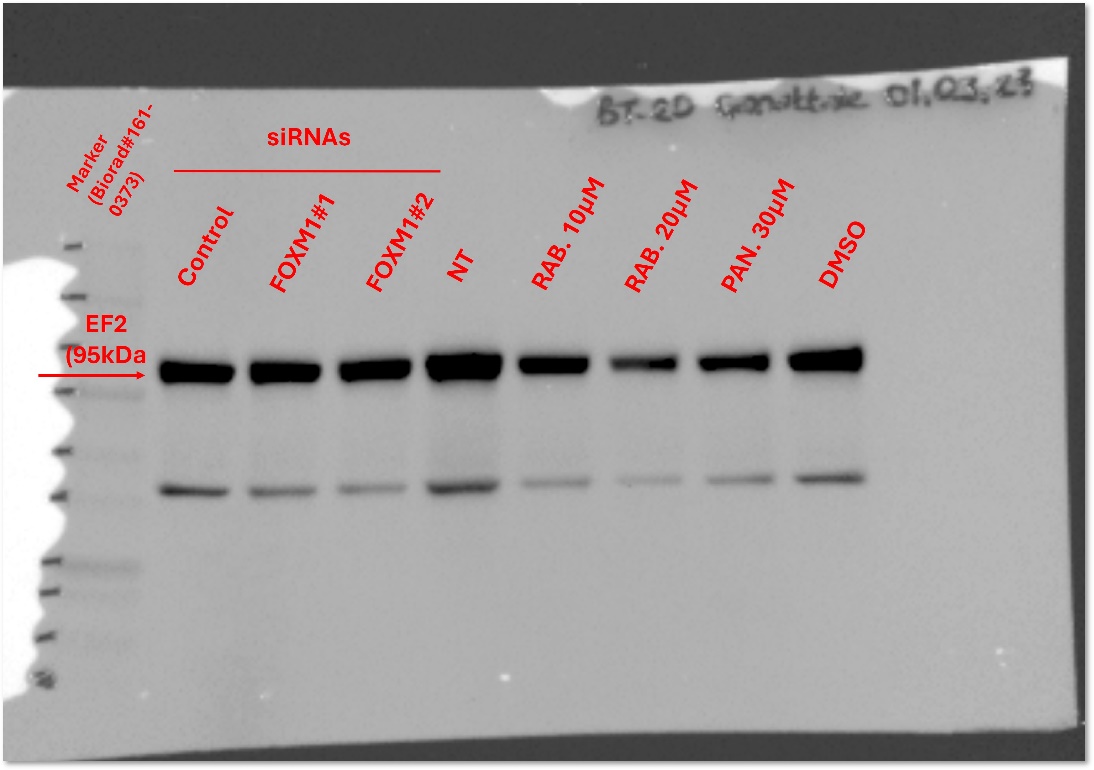


BT-20 EF2


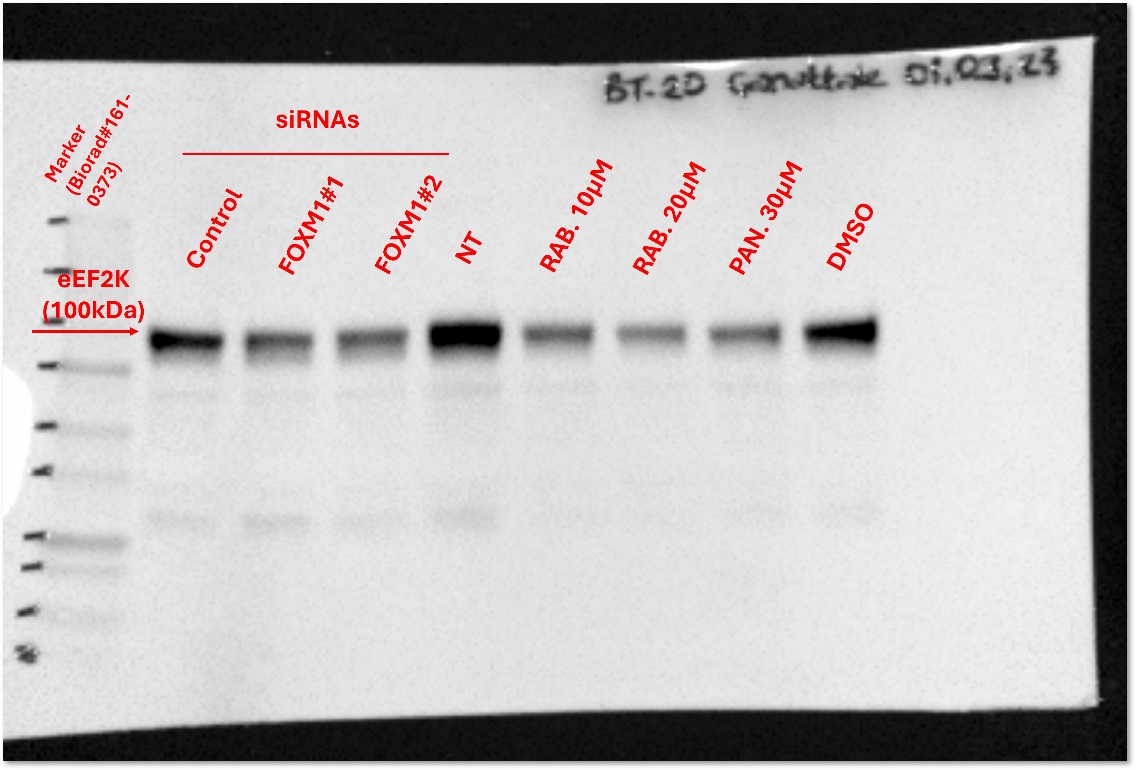


BT-20 EF2K


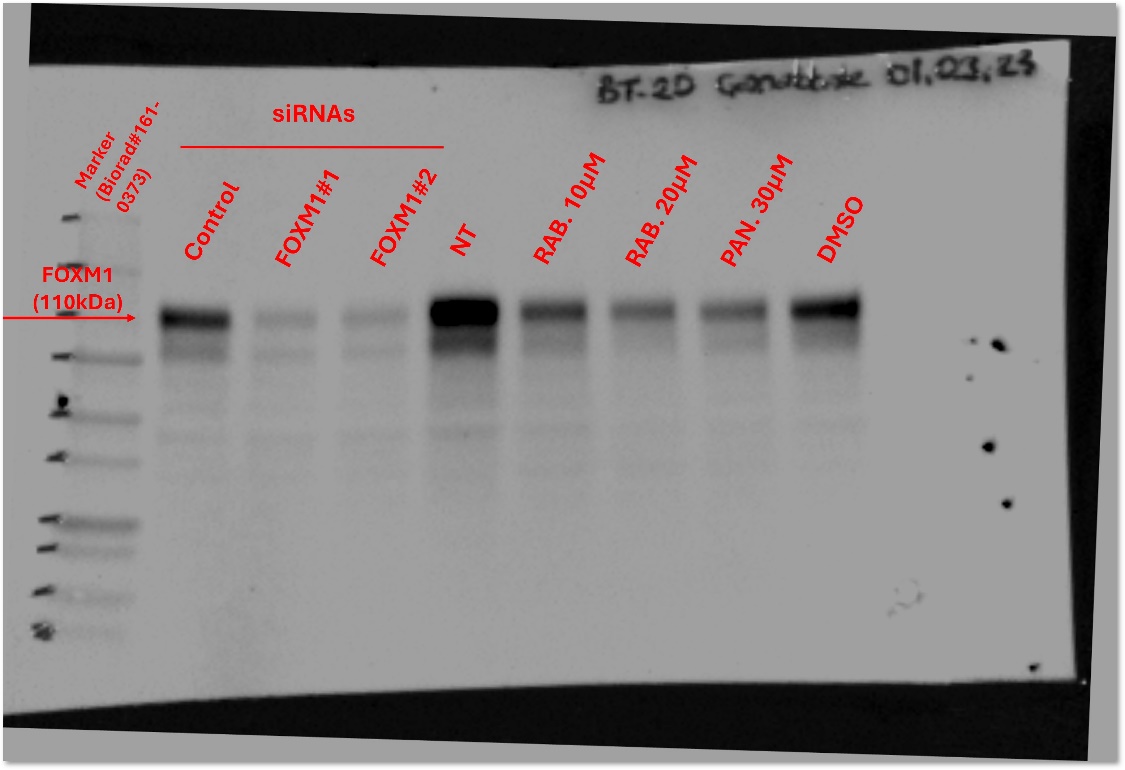


BT-20 FOXM1


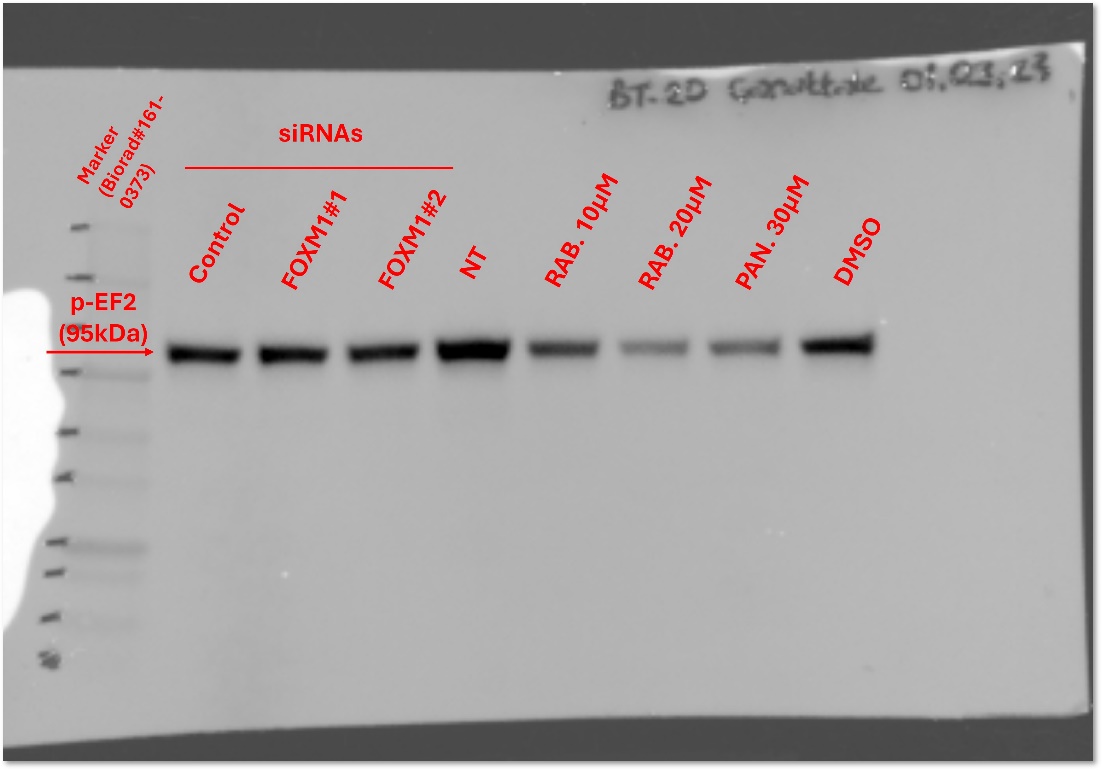


BT-20 p-EF2


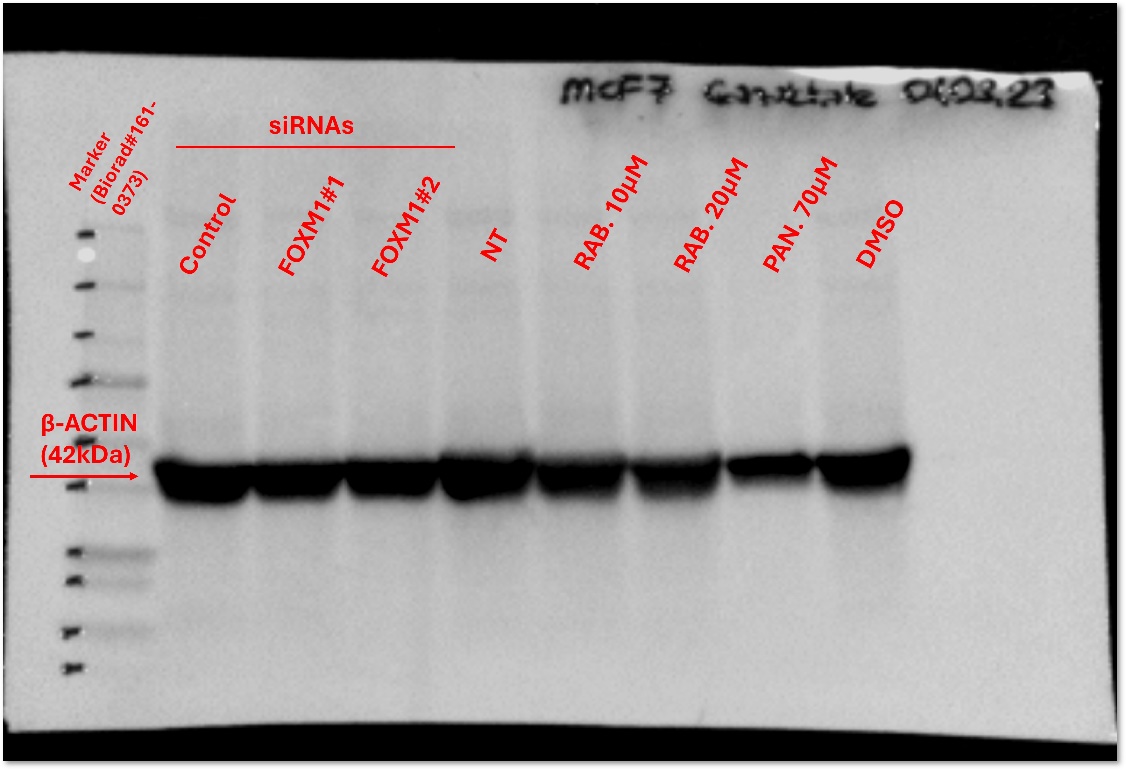


MCF-7 β-actin


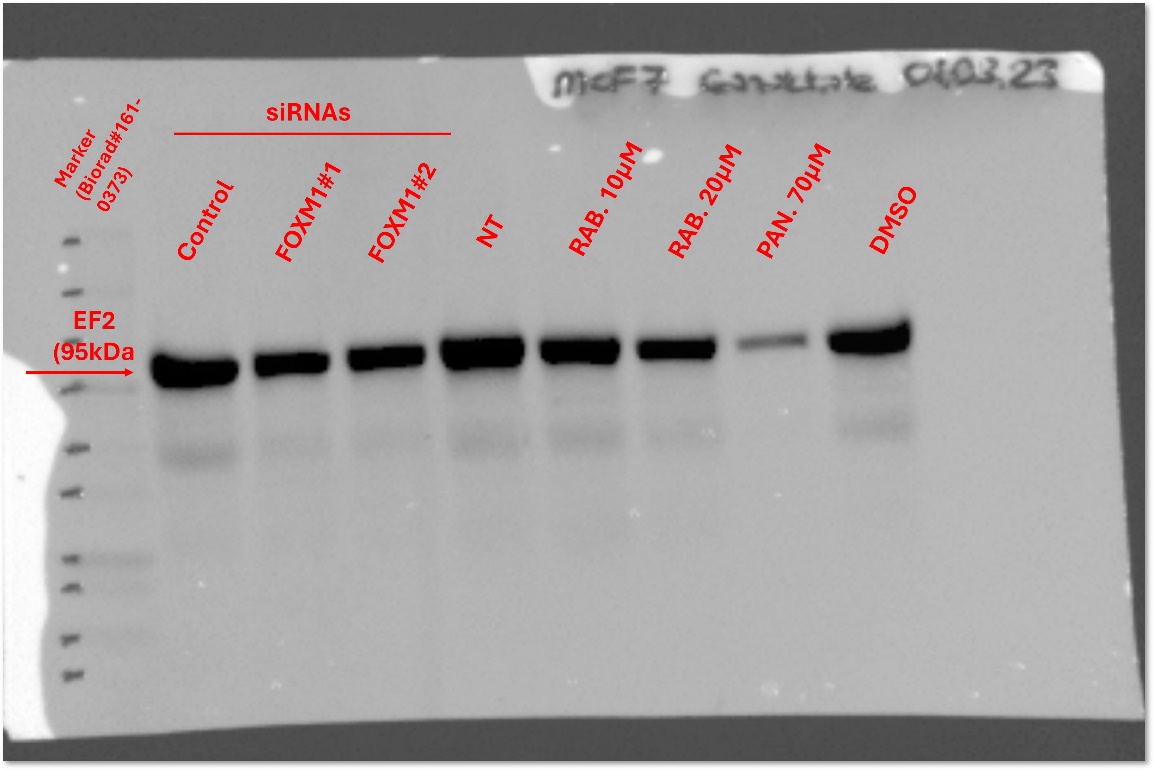


MCF-7 EF2


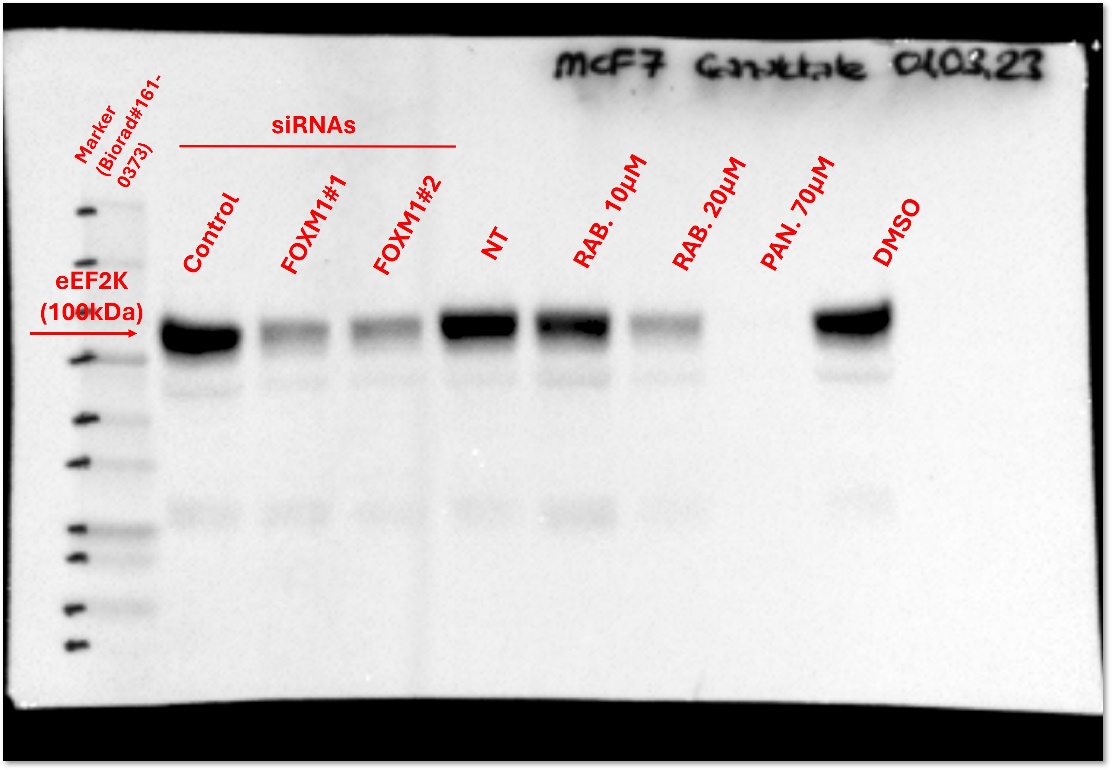


MCF-7 EF2K


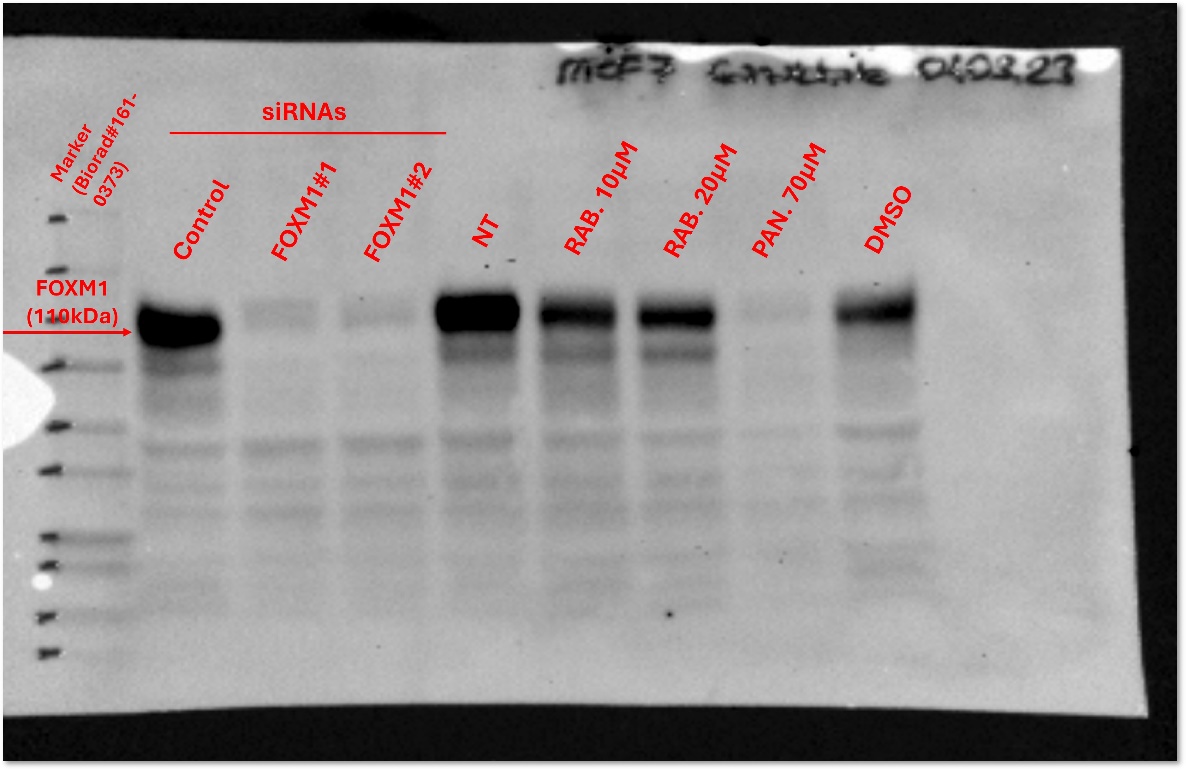


MCF-7 FOXM1


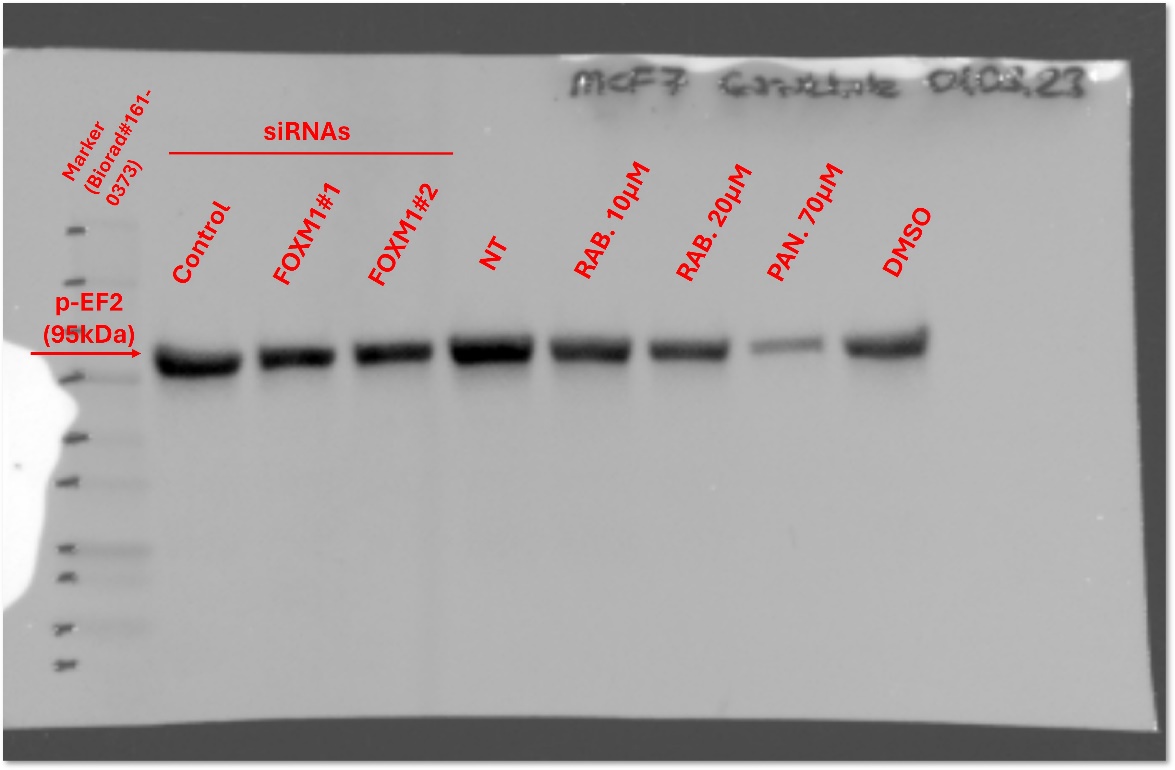


MCF-7 p-EF2
